# Lewis acid-promoted hydrofluorination of alkynyl sulfides to generate α-fluorovinyl thioethers

**DOI:** 10.3762/bjoc.11.205

**Published:** 2015-10-14

**Authors:** Davide Bello, David O'Hagan

**Affiliations:** 1 University of St Andrews, School of Chemistry, North Haugh, St Andrews, Fife, KY16 9ST, UK

**Keywords:** alkynyl sulfides, α-fluorovinyl thioethers, hydrofluorination, Lewis acids, organofluorine

## Abstract

A new method for the preparation of α-fluorovinyl thioethers is reported which involves the hydrofluorination of alkynyl sulfides with 3HF·Et_3_N, a process that requires Lewis acid activation using BF_3_·Et_2_O and TiF_4_. The method gives access to a range of α-fluorovinyl thioethers, some in high stereoselectivity with the *Z*-isomer predominating over the *E*-isomer. The α-fluorovinyl thioether motif has prospects as a steric and electronic mimetic of thioester enols and enolates, important intermediates in enzymatic C–C bond forming reactions. The method opens access to appropriate analogues for investigations in this direction.

## Introduction

**Figure 1:**
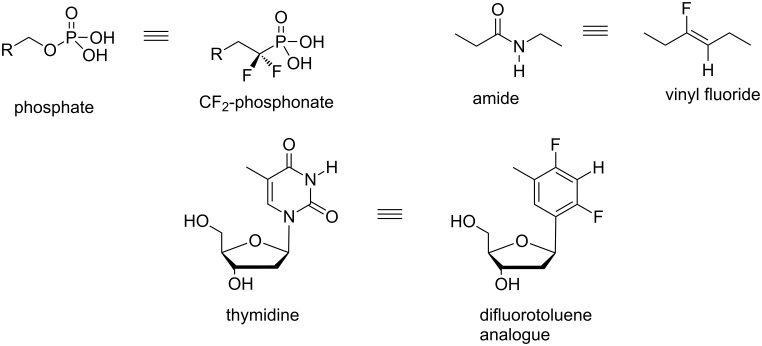
Some spacial and electronic mimetics with fluorine as a design feature [3–6].

Organofluorine compounds have found wide use in tuning the properties of performance compounds in medicinal and materials chemistry [1–2]. Also the electronegativity of fluorine has been used to design and tune steric and electronic mimetics of functional groups for applications in biomolecular chemistry. For example as illustrated in Figure 1, CF_2_-phosphonates became popular mimetics of the phosphate group [3–4], and vinyl fluorides were developed as analogues of the amide bond [5]. Difluorotoluene has proved to be a good spacial mimetic of the thymine base in thymidine, and has been shown to act as a functional and complementary template in enzymatic DNA synthesis [6].

**Figure 2:**
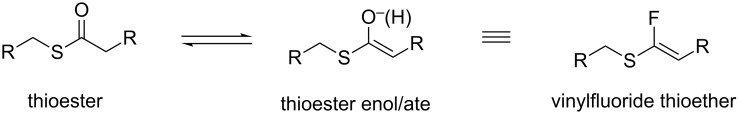
α-Fluorovinyl thioesters offer prospects as thioester enol/ate mimetics [7].

We have recently begun to explore synthesis methods to prepare α-fluorovinyl thioethers, to open up the possibility of exploring this motif as a mimetic for enols and enolates of biochemically relevant thioesters. Thioesters of low molecular weight carboxylic acids are found widely in metabolism, often as their co-enzyme A esters, and they then undergo condensation reactions through enols or enolates to generate C–C bonds typified by the processes of long chain fatty acid biosynthesis. α-Fluorovinyl thioethers, illustrated in Figure 2, have a spatial and electrostatic profile consistent with the potential to mimic these enzyme intermediates.

There is limited literature for preparing such analogues. We have previously described the preparation of α-fluorovinyl thioethers by hydrofluorination of the corresponding alkynyl sulfides using HF·Py [7]; in this article we wish to report an improved synthesis of α-fluoroalkenyl thioethers via Lewis acid-mediated hydrofluorination of alkynyl sulfides, a method which brings us closer to being able to prepare analogues of particular design for enzyme inhibition studies.

## Results and Discussion

Several methods for the synthesis of vinyl thioethers have been reported, including Wittig reactions [8], ionic and radical additions of thiols to alkynes [9] and coupling of 1-alkenyl halides with thiols, among others [10–11]. However, the literature for the preparation of α-fluorovinyl thioethers is somewhat scarce. The only account we are aware of involves the AIBN-promoted thiodesulfonylation of aromatic fluorovinyl sulfones as reported by Wnuk [12], a reaction which works in varying yields and stereoselectivities.

**Scheme 1:**
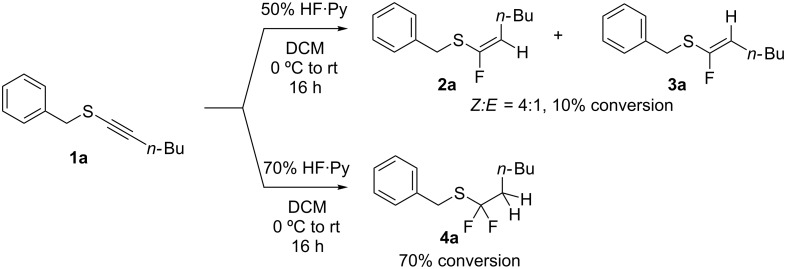
HF·Py mediated hydrofluorinations of **1a**.

Following from our previous experience [7] with terminal acetylene thioethers, we now explore this reaction with alkynyl sulfides. In this regard **1a** [13] was used as a model substrate and was treated with 50% HF·Py in dichloromethane. This, however, resulted in a very poor conversion (~10%) and gave a 4:1 product mixture of the fluorinated products **2a** and **3a** as illustrated in Scheme 1. When 70% HF·Py was employed, up to 70% conversion was achieved, but with over-fluorination to generate only the difluoromethylene thioether **4a** (not isolated).

In view of the lack of control with HF·Py attention turned to triethylamine trihydrogen fluoride (3HF·Et_3_N). This proved unsuccessful presumably as it is a less acidic reagent compared to HF·Py, and thus activation of alkynyl sulfide **1a** was explored by addition of a Lewis acid.

**Scheme 2:**
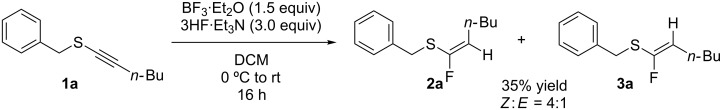
BF_3_·Et_2_O/3HF·Et_3_N mediated hydrofluorination of **1a**.

At this stage we were pleased to find that the use of BF_3_·Et_2_O allowed for a conversion of over 90% of **1a** (16 h at room temperature). However, products **2a** and **3a** were obtained as a 4:1 mixture of *Z*/*E*-isomers, and they could only be isolated in a modest yield (35%) as shown in Scheme 2 and Table 1 (entry 7).

Encouraged by this result, a number of Lewis acids were tested, including SnCl_2_, ZnCl_2_, Sc(OTf)_3_, AuCl·SMe_2_ and B(C_6_F_5_)_3_ (Table 1). The Lewis acids (1.5 equivalents) were added to a mixture of sulfide **1a** and 3HF·Et_3_N (3.0 equivalents) at 0 ºC, but no reactions took place under these conditions. The HBF_4_·SiO_2_ reagent was chosen as a solid phase-supported HBF_4_ equivalent [14]; carrying out the reaction in the presence of this reactant and 3HF·Et_3_N led to complete decomposition of sulfide **1a**.

**Table 1:** Lewis acid screening.

Entry	Lewis acid	HF source	Time	Temp	Conversion	Yield	*Z/E*

1	SnCl_2_	3HF·Et_3_N	16 h	0 °C to rt	0%	–	–
2	ZnCl_2_	3HF·Et_3_N	16 h	0 °C to rt	0%	–	–
3	Sc(OTf)_3_	3HF·Et_3_N	16 h	0 °C to rt	0%	–	–
4	SiO_2_·HBF_4_	3HF·Et_3_N	16 h	0 °C to rt	n.a.^a^	–	–
5	AuCl·SMe_2_	3HF·Et_3_N	16 h	0 °C to rt	0%	–	–
6	B(C_6_F_5_)_3_	3HF·Et_3_N	16 h	0 °C to rt	0%	–	–
7	BF_3_·Et_2_O	3HF·Et_3_N	16 h	0 °C to rt	>90%	35%	4:1
8	TiF_4_	3HF·Et_3_N	16 h	0 °C to rt	70%	42%	4:1

^a^Substrate decomposed.

With TiF_4_ the overall conversion was around 70%, and the hydrofluorinated product could be isolated in an improved yield (42%, 4:1 *Z:E*).

In order to improve the reaction yields, reactions with the BF_3_·Et_2_O/3HF·Et_3_N and TiF_4_/3HF·Et_3_N systems were optimised and the outcomes described in Table 2 and Table 3, respectively. Shorter reaction times (5 h) led to reduced conversions (Table 2, entry 2) and BF_3_·Et_2_O or TiF_4_ are required to be stoichiometric, otherwise the reaction does not occur (Table 2, entry 4) and an excess of BF_3_·Et_2_O over the alkynyl sulfide is required for an improved outcome (Table 2, entry 1).

**Table 2:** Optimisation of BF_3_·Et_2_O/3HF·Et_3_N mediated hydrofluorination.

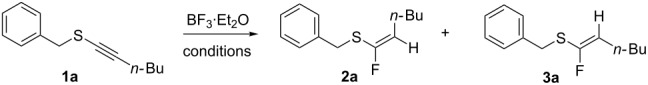							

Entry	BF_3_·Et_2_O (equiv)	3HF·Et_3_N (equiv)	Time	Temp.	Solvent	Conversion	Yield

1	1.5	3.0	16 h	0 °C to rt	DCM	>90%	35%
2	1.5	3.0	5 h	0 °C to rt	DCM	39%	28%
3	1.0	2.0	16 h	0 °C to rt	DCM	>80%	30%
4	0.5	3.0	16 h	0 °C to rt	DCM	–	–
5	1.5	3.0	5 days	0 °C	DCM	20%	–
6	1.5 × 2	3.0	7 h	0 °C	DCM	20%	–
7	1.5	3.0	5 h	40 °C	DCM	>95%	30%
8^a^	1.5	3.0	16 h	0 °C to rt	DCM	70%	28%
9	1.5 × 2	3.0 × 2	21 h	^b^	THF	25%	–
10	1.5	3.0	16 h	0 °C to rt	DCE	<5%	–
11	1.5	3.0	21 h	^c^	DCE	10%	–
12	1.5 x 2	3.0 x 2	21 h	^d^	DCE	n.a.^e^	–

^a^BF_3_·Et_2_O and 3HF·Et_3_N were pre-mixed at 0 °C prior to adding starting material **1a**. ^b^Mixture stirred for 16 hours at room temperature, then heated to 50 °C for 5 hours. ^c^Mixture stirred for 16 hours at room temperature, then stirred under reflux for 5 hours. ^d^Mixture stirred for 5 hours at room temperature, then stirred under reflux for 16 hours. ^e^Substrate decomposed.

The high conversion of **1a** but low product (**2a** and **3a**) isolation is attributed to substrate decomposition. When the reaction is followed by ^19^F NMR (vide infra), the presence of the hydrofluorinated products **2a** and **3a** is obvious and the anion BF_4_^−^, when using BF_3_·Et_2_O, or TiF_6_^2−^ when using TiF_4_ are clearly identifiable. No other fluorinated species are detected, thus it does not appear that products **2a** and **3a** decompose.

A number of attempts were made to improve the yields and reduce starting material decomposition. At low temperatures the reaction is sluggish and conversions are low (~20%), even with prolonged reaction times (5 days, Table 2, entry 5). A second addition of 1.5 equivalents of BF_3_·Et_2_O after a few hours at 0 °C proved ineffective (Table 2, entry 6). On the other hand, warming the mixture to reflux (40 ºC for dichloromethane) allowed for complete conversion in just 5 hours (Table 2, entry 7) although the isolated yield (30%) was relatively modest. Thus heating promotes the reaction but also substrate decomposition. Pre-equilibration of BF_3_·Et_2_O and 3HF·Et_3_N at 0 °C prior to starting material **1a** addition resulted in a 70% conversion and a modest 28% yield (Table 2, entry 8). When tetrahydrofuran or dichloroethane were explored as solvents the conversions were low, even when warming (tetrahydrofuran, Table 2, entry 9, dichloroethane, Table 2, entries 10–12).

For the TiF_4_/3HF·Et_3_N reactions (Table 3) shorter reaction times also afforded lower conversions, and sub-stoichiometric levels of TiF_4_ failed to initiate the reaction. Tetrahydrofuran and dichloroethane at different temperatures were again not useful solvents.

**Table 3:** Optimisation of TiF_4_/3HF·Et_3_N mediated hydrofluorination.

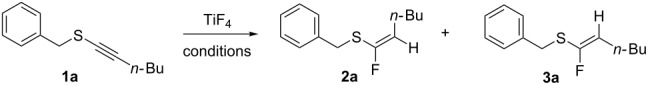							

Entry	TiF_4_ (equiv)	3HF·TEA (equiv)	Time	Temp.	Solvent	Conversion	Yield

1	1.5	3.0	5 h	0 °C to rt	DCM	39%	–
2	1.5	3.0	16 h	0 °C to rt	DCM	>90%	42%
3	0.5	3.0	16 h	0 °C to rt	DCM	–	–
4	1.5	3.0	16 h	0 °C to rt or reflux	THF	–	–
5	1.5	3.0	16 h	0 °C to rt, then reflux	DCE	10%	–

Having optimised the reaction to some extent with substrate **1a**, a range of alkynyl sulfides [15] were now prepared and each individually treated with both hydrofluorination protocols using BF_3_·Et_2_O/3HF·Et_3_N and TiF_4_/3HF·Et_3_N. The results are summarised in Table 4. Cyclohexylethynyl(benzyl)sulfane (**1b**) gave an improved outcome relative to **1a** with higher yields and better stereoselectivity. The BF_3_·Et_2_O reaction furnished an inseparable 9:1 mixture of *Z*-**2b** and *E*-**3b** isomers in 48% yield. When TiF_4_ was used, the reaction showed complete stereoselectivity, affording the *Z*-isomer of **2b** in 55% yield.

**Table 4:** Scope of BF_3_·Et_2_O and TiF_4_-mediated hydrofluorination reaction.

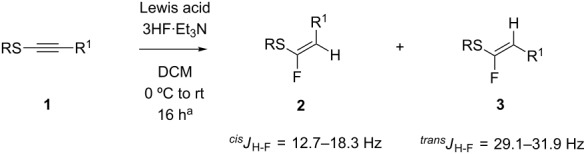			

Substrate	Conversion and yield		Products^a^

**1a** R = Bn R^1^ = *n*-Bu	BF_3_·Et_2_O	>90%, 35% *Z/E* 4:1	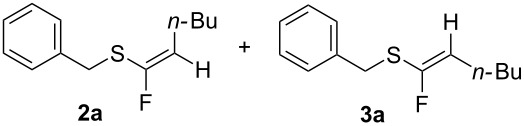
	TiF_4_	70%, 42% *Z/E* 4:1	
**1b** R = Bn R^1^ = Cy	BF_3_·Et_2_O	>90%, 48% *Z/E* 9:1	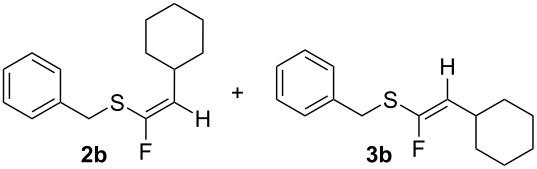
	TiF_4_	80%, 55% *Z* only (**2b**)	
**1c** R = Bn R^1^ = Ph	BF_3_·Et_2_O	60%, 45% *Z* only	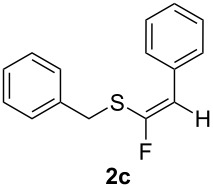
	TiF_4_	>90%, 57% *Z* only	
**1d** R = Cy R^1^ = Ph	BF_3_·Et_2_O	complete, 47% *Z* only	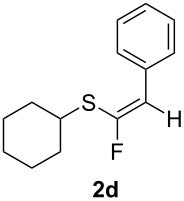
	TiF_4_	>90%, 68% *Z* only	
**1e** R = Ph R^1^ = cyclopropyl	BF_3_·Et_2_O	>80%, 47% *Z/E* 3:2	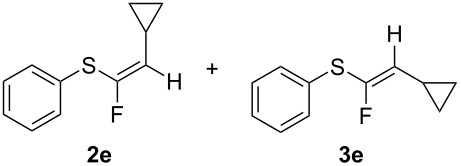
	TiF_4_	90%, 69% *Z/E* 7:3	
**1f** R = Ph R^1^ = *t*-Bu	BF_3_·Et_2_O	80%, 40% *Z* only (contains 2% **4f**)	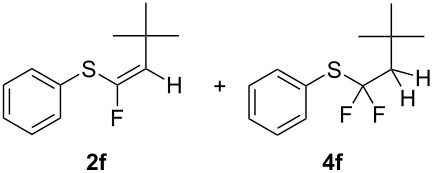
	TiF_4_	>90%, 62% *Z* only (contains 2% **4f**)	
**1g** R = Ph R^1^ = Ph	BF_3_·Et_2_O	75%, 32% *Z* only	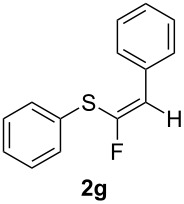
	TiF_4_	80%, 41% *Z* only	
**1h** R = Ph R^1^ = 4-MeOPh	BF_3_·Et_2_O	27%,^b^ 9% *Z* only	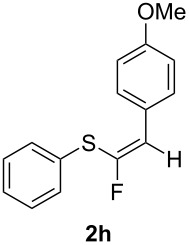
	TiF_4_	35%,^b^ 17% *Z* only	
**1i** R = Ph R^1^ = 4-NO_2_Ph	BF_3_·Et_2_O	90% compound **5** [16], 45% only traces of fluorinated products	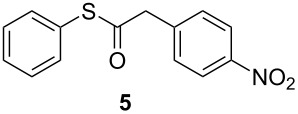
	TiF_4_	15%,^b^ 5% *Z* only	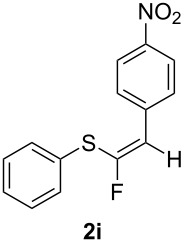
**1j** R = Ph R^1^ = 4-CF_3_Ph	BF_3_·Et_2_O	<5%,^b^ NO products isolated	–
	TiF_4_	<5%,^b^ NO products isolated	

^a^The regiochemistry of all products was determined by NMR analysis. The *Z/E* stereochemistry was determined by calculating the vinyl moieties H–F coupling constants. ^b^Reaction times were 16 hours for all entries except for substrates **1h**, **1i**, and **1j** (7 days).

Replacement of the cyclohexyl moiety with a phenyl ring in **1c** led to a fully stereoselective reaction both with BF_3_·Et_2_O and TiF_4_, giving the *Z*-stereoisomer **2c** in 45% and 57% yields, respectively. We then maintained the phenyl moiety on the alkyne side of the sulfide, and replaced the benzyl group with a cyclohexyl fragment directly connected to the sulfur atom (compound **1d**). This material allowed too for a stereoselective reaction, giving rise to the *Z*-stereoisomer of **2d** in 47% and 68% yields, respectively. At this stage we decided to explore two simple variations of the groups directly connected to the ethynyl moiety, that are, a cyclopropyl group and the bulky *tert*-butyl group. Thus, we reacted cyclopropylethynyl(phenyl)sulfane (**1e**) with BF_3_·Et_2_O, obtaining an inseparable 3:2 mixture of *Z*-**2e** and *E*-**3e** isomers in 47% yield. The reaction with TiF_4_ showed a better stereoselectivity, furnishing a 7:3 *Z/E* mixture in 69% yield.

Interestingly, the reaction of *tert*-butylethynyl(phenyl)sulfane (**1f**) with BF_3_·Et_2_O and TiF_4_, while being completely stereoselective, furnished the *Z-*stereoisomer **2f** in 40% and 62% yields, respectively, along with a 2% of difluorinated compound **4f**. The formation of this byproduct could not be avoided; in fact lower temperatures or shorter reaction times did not change the outcome, and the contaminant **4f** could always be detected (and not removed) from the desired product **2f**.

We were also interested in exploring the electronic effects of *para*-substitution of the phenyl group directly attached to the ethynyl moiety on the reaction outcome; thus we selected compounds **1g–j** and reacted them under our hydrofluorinating conditions. Phenylethynyl(phenyl)sulfane (**1g**) represented the “unactivated” compound in the series. Although the stereoselectivity was complete with the *Z-*isomer of **2g** as the sole product, the yields were unexpectedly low both with BF_3_·Et_2_O and TiF_4_ (32% and 41%, respectively).

We thought that the electron-donating 4-methoxy group would release enough electron density towards the triple bond to increase the yields, and possibly shorten the reaction times. Thus, we prepared compound **1h** and then reacted it with our hydrofluorinating systems; surprisingly, almost no reaction took place during 16 hours, and it was necessary to extend the reaction time to 7 days to obtain the desired product **2h**, which was isolated in 9% yield from the BF_3_·Et_2_O reaction and in 17% when TiF_4_ was employed. It appears that the methoxy group is able to efficiently coordinate the Lewis acid reactants and thus almost prevent the reaction from occurring.

Conversely, and as expected, the 4-nitro group had a detrimental effect on the reaction outcome. When 4-nitrophenyl(ethynyl)sulfane (**1i**) was treated with TiF_4_, it took nearly 7 days to observe some reaction progress, and the desired *Z*-isomer of **2i** could be isolated in only 5% yield. However, when **1i** was reacted with the BF_3_·Et_2_O, the starting material was completely consumed in 16 hours, but only traces of the desired compound **2i** could be detected, with thioester **5** being the main reaction product (45% yield). An explanation for this behaviour can be drawn from the fact that the 4-nitrophenyl group surely must increase the triple bond electrophilicity, hence any trace of water present in the reaction mixture could lead to an intermediate enol thioester which would in turn readily convert to the stable thioester **5**. Nonetheless, ensuring rigorously anhydrous reaction conditions and using fresh BF_3_·Et_2_O could not prevent the formation of **5**, while the same compound was never detected when TiF_4_ was used, even after extended reaction times.

Because of the peculiar reactivity of electron-poor alkynyl sulfide **1i** with respect to BF_3_·Et_2_O and TiF_4_, we decided to carry out a further test with compound **1j**, with the intention of having the 4-trifluoromethylphenyl group removing electron-density from the triple bond, thus possessing a reactivity similar to that of nitro compound **1i**. Surprisingly, compound **1j** was found mostly unreacted after 7 days, and NMR analysis of the crude reaction mixtures did indicate the presence of product **2j** only in traces (<5% conversion). Since **1j** behaved in a similar way both with BF_3_·Et_2_O and TiF_4_, we could only conclude that the formation of thioester **5** from sulfide **1i** was due to some very specific side-reaction promoted by the nitro group, possibly with its participation in the reaction process.

^19^F NMR was used to probe changes in the Lewis acids in the reaction. Ratios of 1:2 Lewis acid:3HF·Et_3_N mixtures in CD_2_Cl_2_ were stirred at room temperature for 2 hours, and the aliquots (0.7 mL) were assayed in Teflon NMR tubes. ^19^F NMR indicated that for each Lewis acid, BF_3_ and TiF_4_, respectively had disappeared, forming the corresponding anions BF_4_^−^ (−150.75 ppm) and [TiF_6_]^2−^ (75.37 ppm), respectively. Broad peaks corresponding to the excess 3HF·Et_3_N reagent were present. BF_4_^−^ is known to be an inherently inert, non-nucleophilic counter ion; in the case of TiF_4_, [TiF_6_]^2−^ was the only species present in solution, and we were unable to detect any penta-coordinated [TiF_5_]^−^ species. It has been reported that an excess of hydrofluoric acid positions the equilibrium between [TiF_5_]^−^ and [TiF_6_]^2−^ in favour of the latter [17]. Moreover [TiF_6_]^2−^ is rather unreactive [18], similar to the BF_4_^−^ anion. We then analysed both reaction mixtures by ^19^F NMR, separately in CD_2_Cl_2_, in the presence of sulfide **1a**, after stirring at room temperature for 2 hours. This showed the presence of **2a** and **3a**, as well anions BF_4_^−^ or [TiF_6_]^2−^ and also an excess 3HF·Et_3_N.

**Scheme 3:**
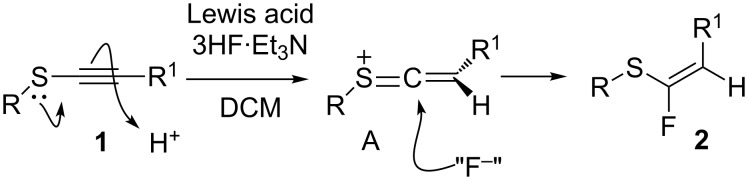
Proposed Lewis acid-mediated hydroflurination of sulfides **1**.

In light of these observations, our working hypothesis is that the Lewis acid acts to increase the acidity of the 3HF·Et_3_N by sequestering fluoride ions as relatively unreactive metal fluorides; thus, the alkynyl sulfides are activated by protonation possibly through an intermediate such as **A** as illustrated in Scheme 3. Such an intermediate would then be susceptible to fluoride ion attack, and progress to the reaction products. The major *cis* stereoselectivity is consistent with the attack of an intermediate such as **A** from the less hindered face, opposite to the R^1^ substituent (Scheme 3).

## Conclusion

In summary, we have developed a mild method for the synthesis of α-fluorovinyl thioethers. The method involves the hydrofluorination of alkynyl sulfides by 3HF·Et_3_N and requires activation using BF_3_·Et_2_O or TiF_4_. The reactions display moderate to good stereoselectivity in favour of the *Z-*hydrofluorination product, and this opens the way forward for making appropriate analogues as potential steric and electronic mimetics of thioester enols and enolates relevant to particular enzymatic transformations.

## Supporting Information

File 1 Experimental part and NMR spectra of synthesised compounds.
