# Supplementary material for: Lewis acid-promoted hydrofluorination of alkynyl sulfides to generate α-fluorovinyl thioethers
Source: Beilstein J Org Chem. 2015 Oct 14;11:1902–9. doi: 10.3762/bjoc.11.205 (PMC4661019; doi:10.3762/bjoc.11.205)
Supplement: File 1 — Experimental part and NMR spectra of synthesised compounds. [file Beilstein_J_Org_Chem-11-1902-s001.pdf]

Supporting Information

for

Lewis acid-promoted hydrofluorination of alkynyl sulfides to generate α-fluorovinyl thioethers

Davide Bello and David O'Hagan*

Address: University of St Andrews, School of Chemistry, North Haugh, St Andrews, Fife, KY16 9ST, UK

Email: David O'Hagan - do1@st-andrews.ac.uk

* Corresponding author

Experimental part and NMR spectra of synthesised compounds

General experimental procedures

All commercially available reagents were purchased from Acros, Alfa Aesar, Fisher Scientific, Fluorochem or Sigma-Aldrich and used without further purification. All glassware was flame-dried and allowed to cool down under high vacuum. Reactions involving the use of $3HF \cdot Et_3N$ or $(HF)_n \cdot$Pyridine reagents, were carried out in Teflon round bottom flasks previously dried overnight in a 160 ºC oven. Dry solvents dichloromethane, diethyl ether and tetrahydrofuran were obtained from the MBraun SPS-800 Solvent Purification System, by passing the solvent through two drying columns under an argon atmosphere. Reaction temperatures of −78 °C to −10 °C were obtained using isopropyl alcohol bath together with LabPlant Refrigerated Immersion Probe. Temperature of 0 °C was obtained using an ice/water bath. Reactions requiring heating or reflux were carried out using a heating block with a contact thermometer.
Thin layer chromatography (TLC) was performed using Merck TLC silica gel 60 F_{254} glass-backed plates. Compounds were visualised by either UV light (254 nm) or by the use of potassium permanganate stain or molybdenum-based stain. Reversed-phase semi-preparative HPLC column chromatography were performed using a Waters 600E multisolvent HPLC system coupled to a Waters 2487 dual wavelength absorbance detector and a Phenomenex Kingsorb® C_{18} 250 × 21.20 mm 5 µm column, eluting with water and acetonitrile, or a Phenomenex Luna® Silica 250 × 21.20 mm 5 µm column, eluting with ethyl acetate and hexane. Retention time values (minutes) are quoted as Rt.

NMR spectra were acquired on Bruker Avance 300 (^{1}H at 300 MHz, ^{13}C at 75 MHz, ^{19}F at 282 MHz) or Bruker Avance II 400 spectrometer (^{1}H at 400 MHz, ^{13}C at 100 MHz, ^{19}F at 376 MHz), or Bruker Avance 500 spectrometer (^{1}H at 500 MHz, ^{13}C at 126 MHz, ^{19}F at 470 MHz). Chemical shifts (δ) are reported in parts per milion (ppm) and are quoted relative to the residual peak of $CDCl_3$. Coupling constants (*J*) are given in Hertz (Hz). ^{13}C NMR and ^{19}F NMR spectra were recorded with ^{1}H decoupling. Signal splitting patterns are described as: s – singlet, br s – broad singlet, d – doublet, dt - doublet of triplets t – triplet, tt – triplet of triplets, m – multiplet.
Mass spectrometric data were acquired by electron impact ionisation (EI), electrospray ionisation (ESI) or chemical ionisation (CI), using Waters Micromass LCT (ESI) or GCT (CI) spectrometers (University of St Andrews), or Thermofisher LTQ Orbitrap XL spectrometer (APCI), Waters GCT Premier (GC–MS EI) and Thermo Scientific Trace-DSQ-II (GC–MS EI) instruments (National Mass Spectrometry Service Centre, Swansea). Values are reported as a ratio of mass to charge (*m*/*z*).

• Experimental section

Benzyl(hex-1-yn-1-yl)sulfane (1a)

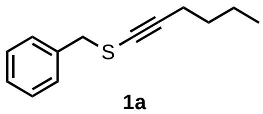

1a

1-Hexyne (2.0 mL, 17.4 mmol, 1.0 equiv) was dissolved in dry diethyl ether (40 mL) under an atmosphere of argon and the resulting mixture cooled to −78 ºC. Butyllithium (1.6 M in hexanes, 10.9 mL, 17.4 mmol, 1.0 equiv) was slowly added dropwise with stirring, and the mixture stirred at −78 ºC for a further 15 minutes. Molecular sulfur (556.8 mg, 17.4 mmol, 1.0 equiv) was then added portionwise at −78 ºC, and the resulting mixture allowed to warm to room temperature and stirred for a further hour. The mixture was then re-cooled to 0 ºC and benzyl bromide (2.1 mL, 17.4 mmol, 1.0 equiv) was added dropwise, and the resulting mixture allowed to warm to room temperature and stirred for 16 hours. The mixture was then treated with a saturated aqueous solution of sodium hydrogen carbonate (20 mL), the layers were separated and the aqueous layer was extracted with diethyl ether (3 × 10 mL). The combined organic layers were dried onto magnesium sulfate, filtered and concentrated under reduced pressure to furnish a yellow oil. Purification by silica gel column chromatography, eluting with hexane, furnished the title compound as a slightly yellow oil (2.7 g, 78% yield). ^{1}H NMR (500 MHz, $CDCl_3$) *δ* 7.37 – 7.28 (m, 5H), 3.91 (s, 2H), 2.29 (t, *J* = 7.0, 2H), 1.51 – 1.41 (m, 2H), 1.40 – 1.32 (m, 2H), 0.90 (t, *J* = 7.3, 3H). These data are in good agreement with the literature values.[1]

Benzyl(cyclohexylethynyl)sulfane (1b)

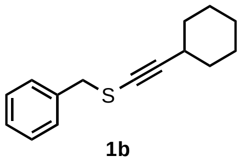

1b

Ethynylcyclohexane (915 µL, 7.0 mmol, 1.0 equiv) was dissolved in dry diethyl ether (15 mL) under an atmosphere of argon, and the resulting mixture cooled to −78 ºC. Butyllithium (1.6 M in hexanes, 4.8 mL, 7.0 mmol, 1.0 equiv) was slowly added dropwise with stirring, and the mixture stirred at −78 ºC for a further 30 minutes. Molecular sulfur (224 mg, 7.0 mmol, 1.0 equiv) was then added portionwise at −78 ºC, and the resulting mixture allowed to warm to room temperature and stirred for a further hour. The mixture was then re-cooled to 0 ºC and benzyl bromide (833 µL, 7.0 mmol, 1.0 equiv) was added dropwise, and the resulting mixture allowed to warm to room temperature and stirred for 16 hours. The mixture was then treated with a saturated aqueous solution of sodium hydrogen carbonate (10 mL), the layers were separated and the aqueous layer was extracted with diethyl ether (3 × 10 mL). The combined organic layers were dried onto magnesium sulfate, filtered and concentrated under reduced pressure to furnish a yellow oil. Purification by silica gel column chromatography, eluting with hexane, furnished the title compound as a clear oil (1.5 g, 91% yield). ^{1}H NMR (500 MHz, $CDCl_3$) δ 7.33 – 7.27 (m, 5H), 3.88 (s, 2H), 2.46 - 2.41 (m, 1H), 1.75 – 1.72 (m, 2H), 1.66 - 1.61 (m, 2H), 1.50 – 1.47 (m, 1H), 1.42 - 1.36 (m, 2H), 1.30 - 1.25 (m, 3H). These data are in good agreement with the literature values.[2]

Benzyl(phenylethynyl)sulfane (1c)

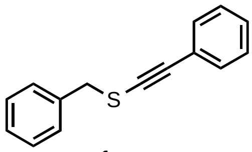

1c

Phenylacetylene (500 µL , 4.55 mmol, 1.0 equiv) was dissolved in dry tetrahydrofuran (10 mL) under and atmosphere of argon, and the resulting mixture cooled to −78 ºC. Butyllithium (1.6 M in hexanes, 2.8 mL, 4.55 mmol, 1.0 equiv) was slowly added dropwise with stirring, and the mixture stirred at −78 ºC for a further 30 minutes. A solution of dibenzyl disulfide (1.12 g, 4.55 mmol, 1.0 equiv) in dry tetrahydrofuran (8 mL) was added dropwise, and the resulting mixture allowed to warm to room temperature and stirred for 3 hours.

At this stage, it was found that the benzyl thiolate anion (BnS^-, generated by displacement upon addition of dibenzyl sulfide to the lithium acetylide) reacted with the forming sulfane **1c**, leading to a bis-sulfide side product and thus reducing the overall yields.[3] This problem was overcome by using 4-nitrobenzyl bromide as a thiolate scavenger. This problem was not observed in the preparation of other sulfanes.

The mixture above was therefore re-cooled to −78 ºC and a solution of 4-nitrobenzyl bromide (982 mg, 4.55 mmol, 1.0 equiv) in dry tetrahydrofuran (5 mL) was added dropwise, and the resulting mixture allowed to slowly reach the room temperature and then stirred for 2 hours. The mixture was then treated with a saturated aqueous solution of sodium hydrogen carbonate (10 mL), the layers were separated and the aqueous layer was extracted with diethyl ether (3 × 10 mL). The combined organic layers were dried onto magnesium sulfate, filtered and concentrated under reduced pressure to furnish a yellow oil. Purification by silica gel column chromatography, eluting with a 10/90 mixture of ethyl acetate and hexane, yielded the title compound as a colourless oil (692 mg, 68% yield). ^{1}H NMR (500 MHz, $CDCl_3$) δ 7.41 - 7.27 (m, 9H), 4.02 (s, 3H). These data are in good agreement with the literature values.[1,3]

Cyclohexyl(phenylethynyl)sulfane (1d)

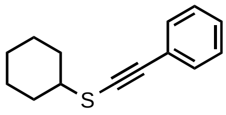

1d

Ethynylbenzene (2.75 mL, 25.1 mmol, 1.1 equiv) was dissolved in dry tetrahydrofuran (50 mL) under an atmosphere of argon, and the resulting mixture cooled to −78 ºC. Butyllithium (1.6 M in hexanes, 15.7 mL, 25.1 mmol, 1.1 equiv) was slowly added dropwise with stirring, and the mixture stirred at −78 ºC for a further 30 minutes. A solution of dicyclohexyl disulfide (5 mL, 22.8 mmol, 1.0 equiv) in dry tetrahydrofuran (10 mL) was then added dropwise, and the resulting mixture allowed to warm to room temperature and stirred for 16 hours. The mixture was then treated with a saturated aqueous solution of sodium hydrogen carbonate (25 mL), the layers were separated and the aqueous layer was extracted with diethyl ether (3 × 20 mL). The combined organic layers were dried onto magnesium sulfate, filtered and concentrated under reduced pressure to furnish a clear oil. Purification by silica gel column chromatography, eluting with hexane, furnished the title compound as a clear oil (3.0 g, 61% yield). ^{1}H NMR (500 MHz, $CDCl_3$) δ 7.43 – 7.41 (m, 2H), 7.31 - 7.28 (m, 3H), 3.00 (tt, J = 11.0, 3.6, 2H), 2.13 - 2.09 (m, 2H), 1.66 - 1.63 (m, 1H), 1.60 - 1.52 (m, 2H), 1.41 - 1.32 (m, 2H), 1.30 - 1.24 (m, 1H). These data are in good agreement with the literature values.[2]

Phenyl(cyclopropylethynyl)sulfane (1e)

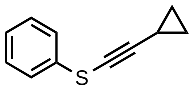

1e

Cyclopropylacetylene (372 µL, 4.4 mmol, 1.1 equiv) was dissolved in dry tetrahydrofuran (15 mL) under and atmosphere of argon, and the resulting mixture cooled to −78 ºC. Butyllithium (1.6 M in hexanes, 2.87 mL, 4.6 mmol, 1.15 equiv) was slowly added dropwise with stirring, and the mixture stirred at −78 ºC for a further 30 minutes. A solution of *S*-phenyl

benzenethiosulfonate (1.0 g, 4.0 mmol, 1.0 equiv) in dry tetrahydrofuran (5 mL) was added dropwise, and the resulting mixture allowed to warm to room temperature and stirred for 16 hours. The mixture was then treated with a saturated aqueous solution of sodium hydrogen carbonate (10 mL), the layers were separated and the aqueous layer was extracted with diethyl ether (3 × 10 mL). The combined organic layers were dried onto magnesium sulfate, filtered and concentrated under reduced pressure to furnish the title compound as a clear oil (570 mg, 74% yield). ^{1}H NMR (500 MHz, $CDCl_3$) δ 7.40 – 7.39 (m, 2H), 7.34 - 7.30 (m, 2H), 7.21 - 7.18 (m, 1H), 1.52 - 1.46 (m, 1H), 0-91 - 0.83 (m, 4H). These data are in good agreement with the literature values.[4]

Phenyl(*t*-butylethynyl)sulfane (1f)

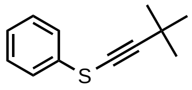

1f

t-Butyl-acetylene (807 µL, 6.6 mmol, 1.1 equiv) was dissolved in dry tetrahydrofuran (20 mL) under and atmosphere of argon, and the resulting mixture cooled to −78 ºC. Butyllithium (1.6 M in hexanes, 4.12 mL, 6.6 mmol, 1.1 equiv) was slowly added dropwise with stirring, and the mixture stirred at −78 ºC for a further 30 minutes. A solution of *S*-phenyl benzenethiosulfonate (1.5 g, 6.0 mmol, 1.0 equiv) in dry tetrahydrofuran (10 mL) was added dropwise, and the resulting mixture allowed to warm to room temperature and stirred for 16 hours. The mixture was then treated with a saturated aqueous solution of sodium hydrogen carbonate (10 mL), the layers were separated and the aqueous layer was extracted with diethyl ether (3 × 10 mL). The combined organic layers were dried onto magnesium sulfate, filtered and concentrated under reduced pressure to furnish a yellow oil. Purification by silica gel column chromatography, eluting with hexane, furnished the title compound as a clear oil (954 mg, 83% yield). ^{1}H NMR (500 MHz, $CDCl_3$) δ 7.40 – 7.37 (m, 2H), 7.34 - 7.30 (m, 2H), 7.21 - 7.17 (m, 1H), 1.33 (s, 9H). These data are in good agreement with the literature values.[5]

Phenyl(phenylethynyl)sulfane (1g)

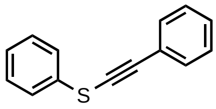

1g

Phenylacetylene (304 µL, 2.8 mmol, 1.1 equiv) was dissolved in dry tetrahydrofuran (7 mL) under and atmosphere of argon, and the resulting mixture cooled to −78 ºC. Butyllithium (1.6 M in hexanes, 1.73 mL, 2.8 mmol, 1.1 equiv) was slowly added dropwise with stirring, and the mixture stirred at −78 ºC for a further 30 minutes. A solution of *S*-phenyl benzenethiosulfonate (630 mg, 2.5 mmol, 1.0 equiv) in dry tetrahydrofuran (3 mL) was added dropwise, and the resulting mixture allowed to warm to room temperature and stirred for 16 hours. The mixture

was then treated with a saturated aqueous solution of sodium hydrogen carbonate (10 mL), the layers were separated and the aqueous layer was extracted with diethyl ether (3 × 5 mL). The combined organic layers were dried onto magnesium sulfate, filtered and concentrated under reduced pressure to furnish the title compound as a slightly yellow oil (426 mg, 81% yield). ^{1}H NMR (500 MHz, $CDCl_3$) δ 7.53 – 7.48 (m, 4H), 7.38 - 7.34 (m, 5H), 7.26 - 7.22 (m, 1H). These data are in good agreement with the literature values.[5]

Phenyl(4-methoxyphenylethynyl)sulfane (1h)

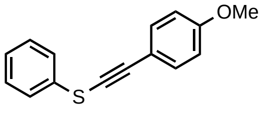

1h

4-Ethynylanisole (560 mg, 4.2 mmol, 1.0 equiv) was dissolved in dry tetrahydrofuran (12 mL) under and atmosphere of argon, and the resulting mixture cooled to −78 ºC. Butyllithium (1.6 M in hexanes, 2.78 mL, 4.45 mmol, 1.05 equiv) was slowly added dropwise with stirring, and the mixture stirred at −78 ºC for a further 30 minutes. A solution of *S*-phenyl benzenethiosulfonate (1.06 g, 4.24 mmol, 1.0 equiv) in dry tetrahydrofuran (5 mL) was added dropwise, and the resulting mixture allowed to warm to room temperature and stirred for 16 hours. The mixture was then treated with a saturated aqueous solution of sodium hydrogen carbonate (10 mL), the layers were separated and the aqueous layer was extracted with diethyl ether (3 × 10 mL). The combined organic layers were dried onto magnesium sulfate, filtered and concentrated under reduced pressure to furnish the title compound as a colourless oil (900 mg, 81% yield). ^{1}H NMR (500 MHz, $CDCl_3$) δ 7.49 - 7.46 (m, 4H), 7.37 – 7.32 (m, 2H), 7.24 - 7.20 (m, 1H), 6.89 - 6.86 (m, 2H), 3.83 (s, 3H). These data are in good agreement with the literature values.[6]

Phenyl(4-nitrophenylethynyl)sulfane (1i)

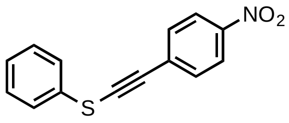

1i

1-Ethynylnitrobenzene (563 mg, 3.83 mmol, 1.0 equiv) was dissolved in dry tetrahydrofuran (15 mL) under and atmosphere of argon, and the resulting mixture cooled to −78 ºC. Sodium bis(trimethylsilyl)amide (1.0 M in tetrahydrofuran, 3.8 mL, 3.8 mmol, 1.0 equiv) was slowly added dropwise with stirring, and the mixture stirred at −78 ºC for a further 15 minutes. A solution of *S*-phenyl benzenethiosulfonate (959 mg, 3.83 mmol, 1.0 equiv) in dry tetrahydrofuran (5 mL) was added dropwise, and the resulting mixture allowed to warm to room temperature and stirred for 5 hours. The mixture was then treated with a saturated aqueous solution of sodium hydrogen carbonate (10 mL), the layers were separated and the aqueous layer was extracted with diethyl ether (3 × 10 mL). The combined organic layers were dried onto

magnesium sulfate, filtered and concentrated under reduced pressure to furnish a brown solid. Purification by silica gel column chromatography, eluting with a 10/90 mixture of ethyl acetate and hexane, furnished the title compound as a deep red solid (650 mg, 67% yield); mp 83 - 85 ºC, ^{1}H NMR (500 MHz, $CDCl_3$) δ 8.22 - 8.19 (m, 2H), 7.60 – 7.58 (m, 2H), 7.51 - 7.49 (m, 2H), 7.42 - 7.38 (m, 2H), 7.31 - 7.28 (m, 1H); ^{13}C NMR (126 MHz, $CDCl_3$) δ 146.93, 131.70, 131.68, 129.86, 129.65, 127.37, 126.91, 123.86, 96.26, 83.24; *m/z* (APCI$^+$) 255.0354. $C_{14}H_9NO_2S$ $[M]^+$ requires 255.0349.

Phenyl(4-trifluoromethylphenylethynyl)sulfane (1j)

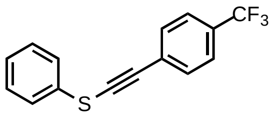

1j

4-Ethynyl-*α,α,α*-trifluorotoluene (500 µL, 3.06 mmol, 1.05 equiv) was dissolved in dry tetrahydrofuran (10 mL) under and atmosphere of argon, and the resulting mixture cooled to −78 ºC. Butyllithium (1.6 M in hexanes, 1.9 mL, 3.06 mmol, 1.05 equiv) was slowly added dropwise with stirring, and the mixture stirred at −78 ºC for a further 30 minutes. A solution of *S*-phenyl benzenethiosulfonate (731 mg, 2.92 mmol, 1.0 equiv) in dry tetrahydrofuran (5 mL) was added dropwise, and the resulting mixture allowed to warm to room temperature and stirred for 3 hours. The mixture was then treated with a saturated aqueous solution of sodium hydrogen carbonate (10 mL), the layers were separated and the aqueous layer was extracted with diethyl ether (3 × 10 mL). The combined organic layers were dried onto magnesium sulfate, filtered and concentrated under reduced pressure to furnish a colourless solid. Purification by silica gel column chromatography, eluting with hexane, furnished the title compound as colourless solid (640 mg, 79% yield); mp 53 - 55 ºC, ^{1}H NMR (500 MHz, $CDCl_3$) δ 7.61 - 7.58 (m, 4H), 7.49 – 7.51 (m, 2H), 7.40 - 7.37 (m, 2H), 7.29 - 7.26 (m, 1H); ^{19}F NMR (470 MHz, $CDCl_3$) - 62.81; ^{13}C NMR (126 MHz, $CDCl_3$) δ 132.25, 131.66, 130.14 (q, $J_{C\text{-}F}$ = 32.4), 129.55, 127.06, 126.80, 126.66, 125.48 (q, $J_{C\text{-}F}$ = 3.9), 124.00 (q, $J_{C\text{-}F}$ = 272.8), 96.57, 79.20; *m/z* (APCI$^+$) 278.0380. $C_{15}H_9F_3S$ $[M]^+$ requires 278.0372.

General procedure for the Lewis acid mediated hydrofluorination of alkynyl sulfides

The alkynyl sulfide (0.5 mmol, 1.0 equiv) was dissolved in dry dichloromethane (2.5 mL) in a PTFE vial maintained under an atmosphere of argon and equipped with a magnetic stirring bar. The resulting mixture was cooled to 0 ºC and triethylamine trihydrofluoride (1.5 mmol, 3.0 equiv) was slowly added with stirring, followed by either boron trifluoride diethyl etherate (0.75 mmol, 1.5 equiv) or titanium tetrafluoride (0.75 mmol, 1.5 equiv). The resulting mixture was allowed to warm to room temperature and stirred for a further 16 hours (unless otherwise stated). The mixture was then diluted with dichloromethane (5 mL) and poured onto a saturated aqueous

solution of sodium hydrogen carbonate (10 mL) and stirred vigorously until the bubbling had ceased. The layers were then separated and the aqueous layer was extracted with dichloromethane (2 x 5 mL), the organic layers combined, dried onto magnesium sulfate and filtered. The resulting solution was concentrated under reduced pressure and purified as described for each specific substance.

(*Z*)-Benzyl(1-fluorohex-1-en-1-yl)sulfane (2a) and (*E*)-benzyl(1-fluorohex-1-en-1-yl)sulfane (3a)

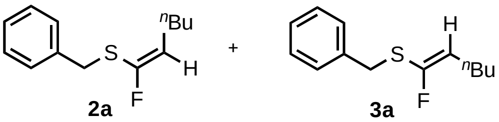

Purification by silica gel column chromatography, eluting with hexane, furnished the desired α-fluorovinyl thioether as a 4:1 inseparable mixture of the *Z* (**2a**) and *E* (**3a**) stereoisomers, respectively, in 35% yield (using boron trifluoride diethyl etherate as the Lewis Acid) and 42% yield (using titanium tetrafluoride as the Lewis Acid).

An analytical sample of the inseparable mixture of the *Z* and *E* isomers was obtained by HPLC purification using a reverse phase HPLC silica column (see the General Experimental Procedures above) with a 10 mL/min isocratic flow of 15/85 water/acetonitrile.

Mixture of compounds obtained as a clear oil. Rt = 9.7; ^{1}H NMR (400 MHz, $CDCl_3$) δ 7.33 - 7.22 [m, 5H (*Z* isomer) + 1.6H (*E* isomer)], 5.48 (dt, $J_{H\text{-}F}$ = 15.7, $J_{H\text{-}H}$ = 7.8, 1H, *Z* isomer), 5.00 (dt, $J_{H\text{-}F}$ = 31.9, $J_{H\text{-}H}$ = 7.8, 0.28H, *E* isomer), 3.90 (s, 2H, *Z* isomer), 3.89 (s, 0.56H, *E* isomer), 2.09 - 2.03 (m, 0.56H, *E* isomer), 1.91 - 1.85 (m, 2H, *Z* isomer), 1.27 - 1.21 (m, 1H, *E* isomer), 1.17-1.03 (m, 4H, *Z* isomer), 0.86 (t, *J* = 6.9, 0.8H, *E* isomer), 0.80 (t, *J* = 6.9, 3H, *Z* isomer); ^{19}F NMR (376 MHz, $CDCl_3$) δ - 88.99 (*Z* isomer), - 94.07 (*E* isomer); ^{13}C NMR (100 MHz, $CDCl_3$) δ 152.59 (d, $J_{C\text{-}F}$ = 286.2, *Z* isomer), 151.67 (d, $J_{C\text{-}F}$ = 291.2, *E* isomer), 137.55, 137.36, 129.00, 128.52, 127.31, 118.95 (d, $J_{C\text{-}F}$ = 20.1, *Z* isomer), 117.63 (d, $J_{C\text{-}F}$ = 23.2, *E* isomer), 37.05, 36.42, 31.37, 31.02, 27.01 (d, $J_{C\text{-}F}$ = 2.9), 24.99, 22.09, 22.06, 13.87; *m/z* (TOF MS EI+) 224.1034. $C_{13}H_{17}FS$ [M] requires 224.1035.

(*Z*)-Benzyl(2-cyclohexyl-1-fluorovinyl)sulfane (2b) and (*E*)-benzyl(2-cyclohexyl-1-fluorovinyl)sulfane (3b)

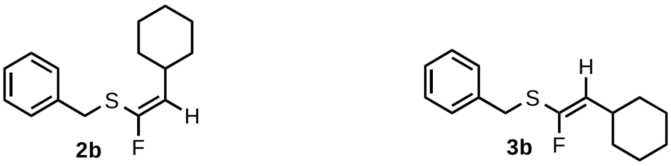

Purification by silica gel column chromatography, eluting with hexane, furnished the desired α-fluorovinyl thioether as a 9:1 mixture of the *Z* (**2b**) and *E* (**3b**) stereoisomers, respectively, in 48% yield using boron trifluoride diethyl etherate as the Lewis Acid.

Using titanium tetrafluoride as the Lewis Acid afforded, after purification by silica gel column chromatography, eluting with hexane, the pure *Z*-α-fluorovinyl thioether **2b**.

Analytical samples of both the *Z* and *E* isomers were obtained by HPLC purification using a normal phase HPLC silica column (see the General Experimental Procedures above) with a 10 mL/min isocratic flow of hexane.

Z isomer (**2b**) obtained as a clear oil. Rt = 21.44; ^{1}H NMR (500 MHz, $CDCl_3$) δ 7.35 - 7.25 (m, 5H), 5.37 (dd, $J_{H\text{-}F}$ = 15.8, $J_{H\text{-}H}$ = 9.8, 1H), 3.92 (s, 2H), 2.05 - 1.97 (m, 1H), 1.63 - 1.57 (m, 3H), 1.30 - 1.27 (m, 2H), 1.21 - 1.08 (m, 3H), 0.96 - 0.88 (m, 2H); ^{19}F NMR (470 MHz, $CDCl_3$) δ - 90.71; ^{13}C NMR (125 MHz, $CDCl_3$) δ 151.41 (d, $J_{C\text{-}F}$ = 290.1), 137.63, 129.05, 128.53, 127.33, 123.57 (d, $J_{C\text{-}F}$ = 20.5), 37.13 (d, $J_{C\text{-}F}$ = 2.8), 36.57, 32.95 (d, $J_{C\text{-}F}$ = 2.1), 25.89, 25.71; *m/z* (TOF MS EI+) 250.1192. $C_{15}H_{19}FS$ [M] requires 250.1192.

E isomer (**3b**) obtained as a clear oil. Rt = 23.39; ^{1}H NMR (500 MHz, $CDCl_3$) δ 7.32 - 7.28 (m, 2H), 7.25 - 7.23 (m, 3H), 4.85 (dd, $J_{H\text{-}F}$ = 31.9, $J_{H\text{-}H}$ = 9.2, 1H), 3.88 (s, 2H), 2.43 - 2.36 (m, 1H), 1.65 - 1.56 (m, 5H), 1.29 - 1.21 (m, 2H), 1.16 - 1.10 (m, 1H), 0.99 - 0.91 (m, 2H); ^{19}F NMR (470 MHz, $CDCl_3$) δ - 93.79; ^{13}C NMR (125 MHz, $CDCl_3$) δ 150.33 (d, $J_{C\text{-}F}$ = 293.7), 137.34, 129.12, 128.54, 127.31, 124.93 (d, $J_{C\text{-}F}$ = 20.3), 37.14, 35.03, 32.61, 25.99, 25.73; *m/z* (TOF MS EI+) 250.1191. $C_{15}H_{19}FS$ [M] requires 250.1192.

(*Z*)-Benzyl(2-phenyl-1-fluorovinyl)sulfane (2c)

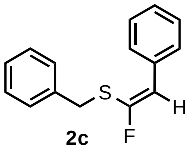

2c

Purification by silica gel column chromatography, eluting with hexane, furnished the desired *Z*-α-fluorovinyl thioether (**2c**) as a clear oil in 45% yield using boron trifluoride diethyl etherate as the Lewis Acid, and in 57% yield using titanium tetrafluoride as the Lewis Acid.

^{1}H NMR (300 MHz, $CDCl_3$) δ 7.35 - 7.20 (m, 10H), 6.56 (d, $J_{H\text{-}F}$ = 17.7, 1H), 4.09 (d, $J_{H\text{-}F}$ = 0.5, 2H); ^{19}F NMR (282 MHz, $CDCl_3$) δ - 83.09; ^{13}C NMR (100 MHz, $CDCl_3$) δ 154.21 (d, $J_{C\text{-}F}$ = 295.0), 136.85, 132.77 (d, $J_{C\text{-}F}$ = 7.1), 129.00, 128.7 (d, $J_{C\text{-}F}$ = 2.9), 128.72, 128.28, 127.61, 127.43, 116.08 (d, $J_{C\text{-}F}$ = 30.4), 36.27 (d, $J_{C\text{-}F}$ = 1.3).

(*Z*)-Cyclohexyl(1-fluoro-2-phenylvinyl)sulfane (2d)

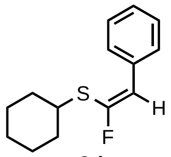

2d

Purification by silica gel column chromatography, eluting with hexane, furnished the desired *Z*-α-fluorovinyl thioether (**2d**) as a clear oil in 47% yield using boron trifluoride diethyl etherate as the Lewis Acid, and in 68% yield using titanium tetrafluoride as the Lewis Acid.

^{1}H NMR (400 MHz, $CDCl_3$) δ 7.54 - 7.51 (m, 2H), 7.35 - 7.31 (m, 2H), 7.26 - 7.22 (m, 1H), 6.58 (d, $J_{H\text{-}F}$ = 18.3, 1H), 3.31 - 3.23 (m, 1H), 2.05 - 2.01 (m, 2H), 1.78 - 1.74 (m, 2H), 1.64 - 1.59 (m, 1H), 1.49 - 1.22 (m, 5H); ^{19}F NMR (376 MHz, $CDCl_3$) δ - 78.10; ^{13}C NMR (125 MHz, $CDCl_3$) δ 154.61 (d, $J_{C\text{-}F}$ = 293.9), 133.14 (d, $J_{C\text{-}F}$ = 10.1), 128.9 (d, $J_{C\text{-}F}$ = 2.3), 128.34, 127.41, 116.49 (d, $J_{C\text{-}F}$ = 33.7), 45.37, 33.53, 26.12, 25.67; *m/z* (TOF MS EI+) 236.1040. $C_{14}H_{17}FS$ [M] requires 236.1035.

(*Z*)-(2-Cyclopropyl-1-fluorovinyl)(phenyl)sulfane (2e) and (*E*)-(2-cyclopropyl-1-fluorovinyl)(phenyl)sulfane (3e)

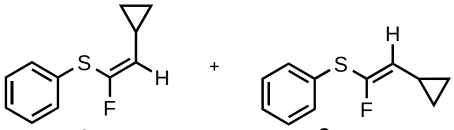

2e **3e**

Purification by silica gel column chromatography, eluting with hexane, furnished the desired α-fluorovinyl thioether as a 3:2 inseparable mixture of the *Z* (**2e**) and *E* (**3e**) stereoisomers, respectively, in 47% yield when using boron trifluoride diethyl etherate as the Lewis Acid.

Using titanium tetrafluoride as the Lewis Acid afforded, after purification by silica gel column chromatography, eluting with hexane, the α-fluorovinyl thioether as a 7:3 inseparable mixture of the *Z* (**2e**) and *E* (**3e**) stereoisomers, respectively, in 69% yield.

An analytical sample of the inseparable mixture of the *Z* and *E* isomers was obtained by HPLC purification using a normal phase HPLC silica column (see the General Experimental Procedures above) with a 10 mL/min isocratic flow of hexane.

Mixture of compounds obtained as a clear oil. Rt = 16.8. ^{1}H NMR (400 MHz, $CDCl_3$) δ 7.42 - 7.22 [m, 5H (*Z* isomer) + 2.5H (*E* isomer)], 5.25 (dd, $J_{H\text{-}F}$ = 12.7, $J_{H\text{-}H}$ = 9.7, 1H, *Z* isomer), 4.95 (dd, $J_{H\text{-}F}$ = 29.1, $J_{H\text{-}H}$ = 7.8, 0.5H, *E* isomer), 1.84 - 1.71 (m, 1H *Z* isomer + 0.5 *E* isomer), 0.89 - 0.82 (m, 2H *Z* isomer + 1H *E* isomer), 0.52 - 0.46 (m, 2H *Z* isomer + 1H *E* isomer); ^{19}F NMR (376 MHz, $CDCl_3$) δ - 89.65 (*Z* isomer), - 97.67 (*E* isomer); ^{13}C NMR (100 MHz, $CDCl_3$) δ

150.36 (d, $J_{C\text{-}F}$ = 290.1, *Z* isomer), 149.9 (d, $J_{C\text{-}F}$ = 297.6, *E* isomer), 133.66 (d, $J_{C\text{-}F}$ = 4.9, *E* isomer), 133.01 (d, $J_{C\text{-}F}$ = 4.4, *Z* isomer), 129.35, 129.29, 128.80, 128.60, 127.05, 126.01 (d, $J_{C\text{-}F}$ = 19.6, *E* isomer), 124.98 (d, $J_{C\text{-}F}$ = 26.7, *Z* isomer), 9.97 (d, $J_{C\text{-}F}$ = 3.2, *Z* isomer), 8.76 (d, $J_{C\text{-}F}$ = 2.4, *E* isomer), 7.38, 7.02; *m/z* (TOF MS EI+) 194.0566. $C_{11}H_{11}FS$ [M] requires 194.0565.

(*Z*)-(1-Fluoro-3,3-dimethylbut-1-en-1-yl)(phenyl)sulfane (2f) and (1,1-difluoro-3,3-dimethylbutyl)(phenyl)sulfane (4f)

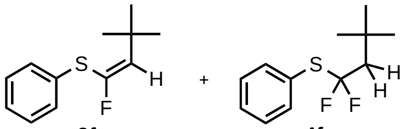

2f **4f**

Purification by silica gel column chromatography, eluting with hexane, furnished the desired *Z*-α-fluorovinyl thioether **2f** contaminated with the difluorinated compound **4f** (ca. 2%), in 40% yield when using boron trifluoride diethyl etherate as the Lewis Acid, and in 62% yield when using titanium tetrafluoride as the Lewis Acid.

Mixture of compounds obtained as a clear oil. ^{1}H NMR (500 MHz, $CDCl_3$) δ 7.33 - 7.31 (m, 5H, **2f**), 7-28 - 7.24 (m, 2H, **2f**), 7.20 - 7.16 (m, 1H, **2f**), 5.85 (d, $J_{H\text{-}F}$ = 19.0, **2f**), 2.08 (t, $J_{H\text{-}F}$ = 16.9, 0.02H, **4f**) 1.16 (s, 9H, **2f**); ^{19}F NMR (470 MHz, $CDCl_3$) δ - 70.10 (**4f**), - 82.32 (**2f**); ^{13}C NMR (125 MHz, $CDCl_3$) δ 150.300 (d, $J_{C\text{-}F}$ = 292.1), 132.55 (d, $J_{C\text{-}F}$ = 3.1), 129.58 (d, $J_{C\text{-}F}$ = 22.5), 129.34, 129.92, 127.10, 30.86, 30.53, *m/z* (TOF MS EI+) 210.0882. $C_{12}H_{15}FS$ [M] requires 210.0878.

(*Z*)-Phenyl(1-fluoro-2-phenylvinyl)sulfane (2g)

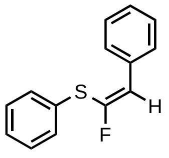

2g

Purification by silica gel column chromatography, eluting with hexane, furnished the desired *Z*-α-fluorovinyl thioether (**2g**) as a clear oil in 32% yield using boron trifluoride diethyl etherate as the Lewis Acid, and in 41% yield using titanium tetrafluoride as the Lewis Acid.

An analytical sample of **2g** was obtained by HPLC purification using a normal phase HPLC silica column (see the General Experimental Procedures above) with a 10 mL/min isocratic flow of 2/98 ethyl acetate/hexane.

Rt = 10.5; ^{1}H NMR (400 MHz, $CDCl_3$) δ 7.57 - 7.55 (m, 2H), 7.46 - 7.43 (m, 2H), 7.37 - 7.28 (m, 6H), 6.78 (d, $J_{H\text{-}F}$ = 16.2, 1H); ^{19}F NMR (376 MHz, $CDCl_3$) δ - 80.34; ^{13}C NMR (125 MHz, $CDCl_3$) δ 152.49 (d, $J_{C\text{-}F}$ = 296.1), 132.50 (d, $J_{C\text{-}F}$ = 9.2), 131.27 (d, $J_{C\text{-}F}$ = 3.2), 129.95, 129.48, 128.96 (d, $J_{C\text{-}F}$ = 3.5), 128.54, 128.10, 127.75, 118.79 (d, $J_{C\text{-}F}$ = 31.7); *m/z* (APCI+) 229.0483. $C_{14}H_{10}FS$ $[M\text{-}H]^+$ requires 229.0482.

(*Z*)-(1-fluoro-2-(4-methoxyphenyl)vinyl)(phenyl)sulfane (2h)

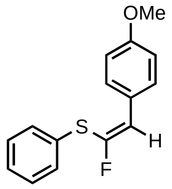

2h

The reaction mixture was stirred at room temperature for **7 days** in order to observe the formation of the desired product. Purification by silica gel column chromatography, eluting with hexane, furnished the *Z*-α-fluorovinyl thioether **2h** as a clear oil in 9% yield using boron trifluoride diethyl etherate as the Lewis Acid, and in 17% yield using titanium tetrafluoride as the Lewis Acid.

An analytical sample of **2h** was obtained by HPLC purification using a reverse phase HPLC silica column (see the General Experimental Procedures above) with a 10 mL/min isocratic flow of 15/85 water/acetonitrile.

Rt = 12.7; ^{1}H NMR (500 MHz, $CDCl_3$) δ 7.54 - 7.51 (m, 2H), 7.44 - 7.42 (m, 2H), 7.34 - 7.31 (m, 2H), 7.28 - 7.25 (m, 2H), 6.90 - 6.87 (m, 2H), 6.77 (d, $J_{H\text{-}F}$ = 16.3, 1H), 6.82 (s, 3H); ^{19}F NMR (470 MHz, $CDCl_3$) δ - 82.35; ^{13}C NMR (125 MHz, $CDCl_3$) δ 159.52, 150.84 (d, $J_{C\text{-}F}$ = 289.6), 131.76 (d, $J_{C\text{-}F}$ = 3.1), 130.25 (d, $J_{C\text{-}F}$ = 3.3), 129.47, 129.45, 127.52, 124.77 (d, $J_{C\text{-}F}$ = 8.3), 119.10 (d, $J_{C\text{-}F}$ = 32.1), 114.01, 55.43; *m/z* (TOF MS EI+) 260.0670. $C_{15}H_{13}FOS$ [M] requires 260.0671.

(*Z*)-(1-fluoro-2-(4-nitrophenyl)vinyl)(phenyl)sulfane (2i)

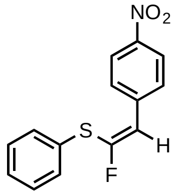

2i

The reaction mixture was stirred at room temperature for **7 days** in order to observe the formation of the desired product. Purification by silica gel column chromatography, eluting with hexane, furnished the *Z*-α-fluorovinyl thioether **2i** as a waxy solid in 5% yield using titanium tetrafluoride as the Lewis Acid.

An analytical sample of **2i** was obtained by HPLC purification using a reverse phase HPLC silica column (see the General Experimental Procedures above) with a 10 mL/min isocratic flow of 15/85 water/acetonitrile.

Rt = 11.7; ^{1}H NMR (300 MHz, $CDCl_3$) δ 8.24 - 8.18 (m, 2H), 7.73 - 7.68 (m, 2H), 7.48 - 7.45 (m, 2H), 7.40 - 7.33 (m, 3H), 6.73 (d, $J_{H\text{-}F}$ = 16.1, 1H), 6.82 (s, 3H); ^{19}F NMR (282 MHz, $CDCl_3$) δ -

74.16; ^{13}C NMR (125 MHz, $CDCl_3$) δ 156.15 (d, $J_{C\text{-}F}$ = 301.83), 139.51 (d, $J_{C\text{-}F}$ = 9.7), 131.67, 131.15, 129.70, 129.51 (d, $J_{C\text{-}F}$ = 3.2), 128.65, 124.08, 123.84, 115.52 (d, $J_{C\text{-}F}$ = 34.2).

Benzyl(hex-1-yn-1-yl)sulfane (1a)

10032013-23-doh-db44-F
1H Observe
DB262_23-50

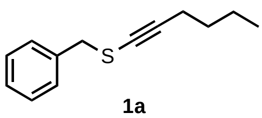

1a

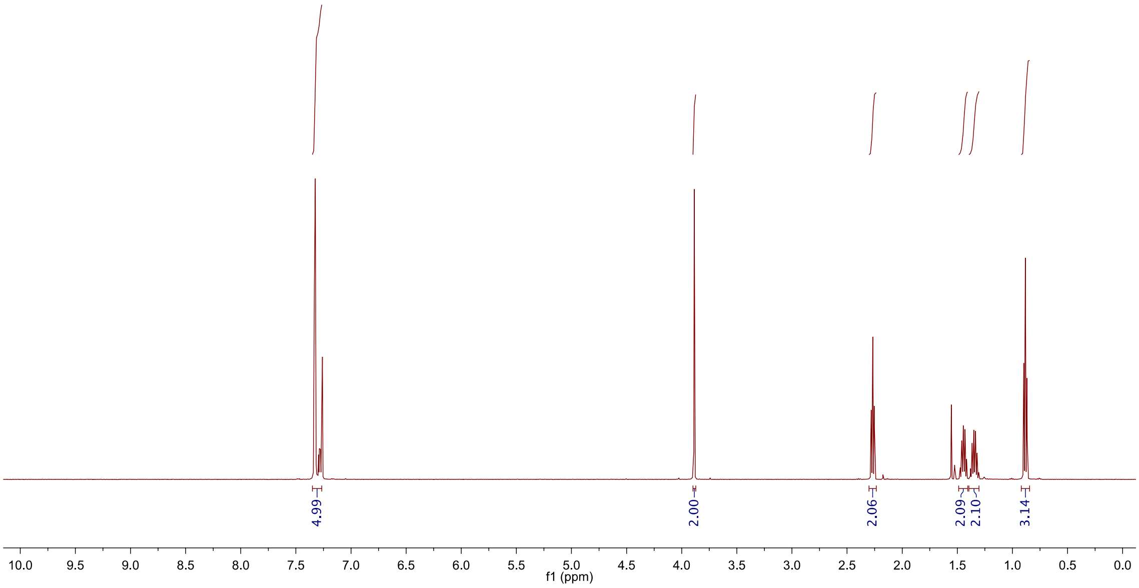

Benzyl(cyclohexylethynyl)sulfane (1b)

02062015-33-doh-db44-A
1H Observe
DB464_15-21

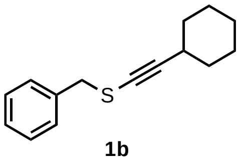

1b

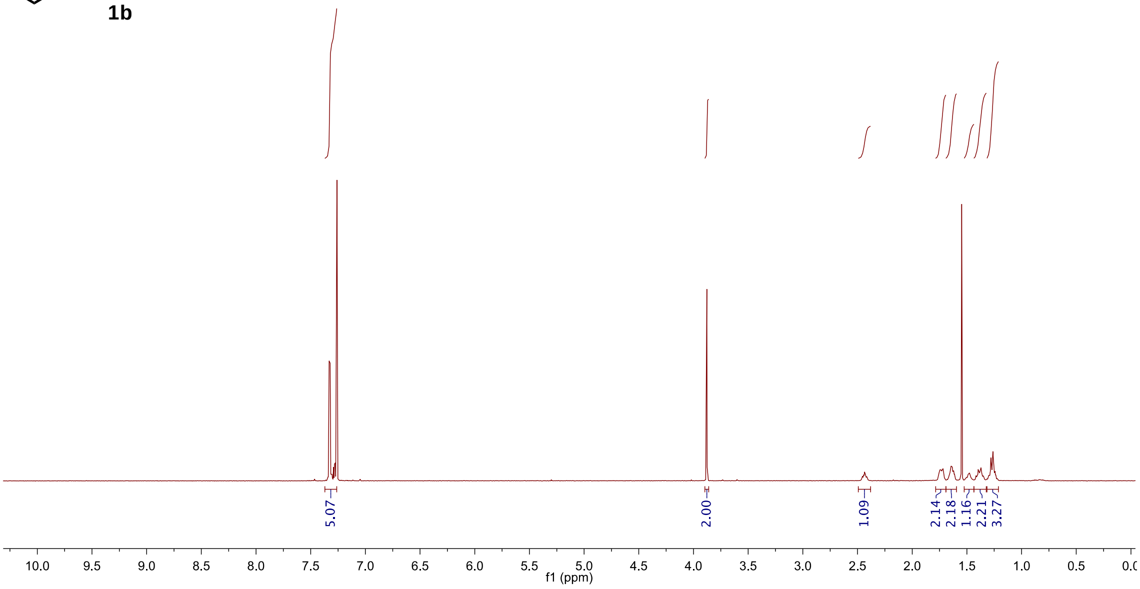

Benzyl(phenylethynyl)sulfane (1c)

03122015-10-doh-db44-R
1H Observe
DB526_12-14

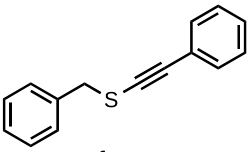

1c

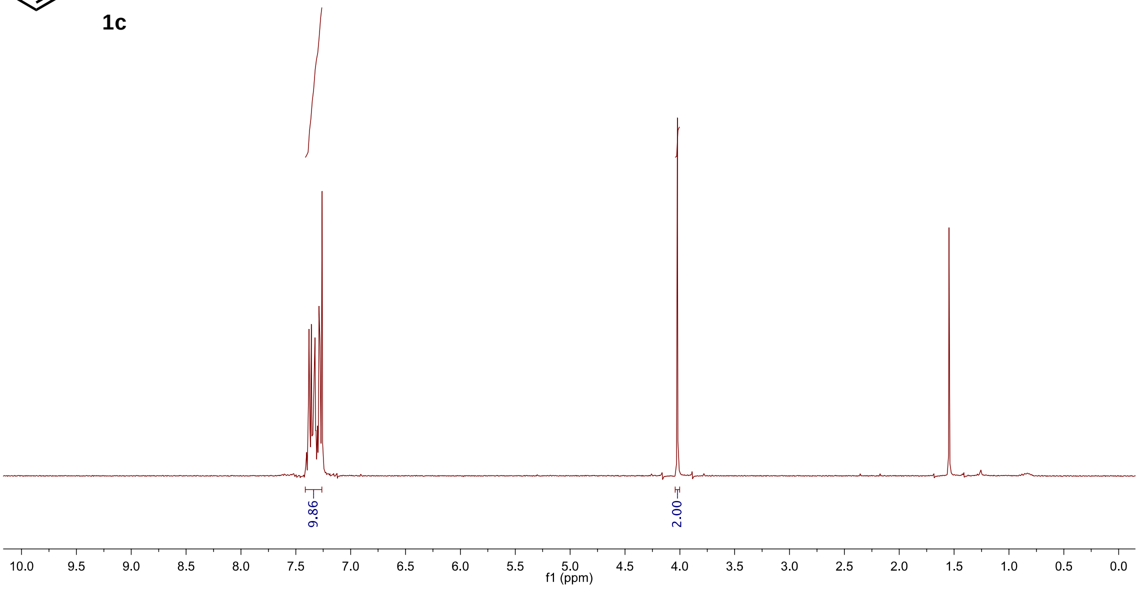

Cyclohexyl(phenylethynyl)sulfane (1d)

01222015-6-doh-db44-A
1H Observe
DB492-24

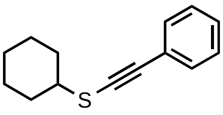

1d

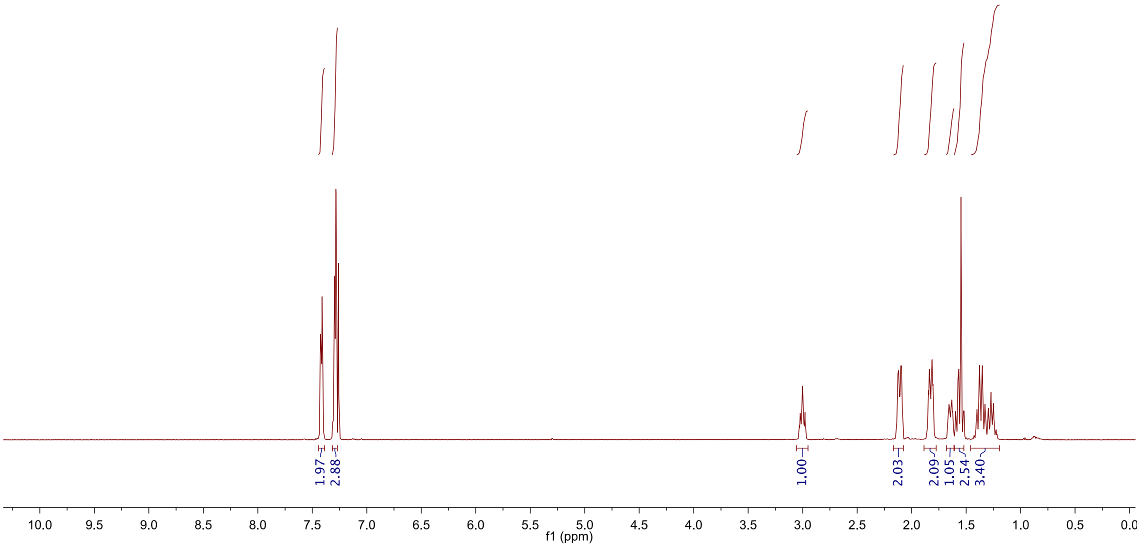

Phenyl(cyclopropylethynyl)sulfane (1e)

02062015-37-doh-db44-A
1H Observe
DB505

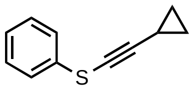

1e

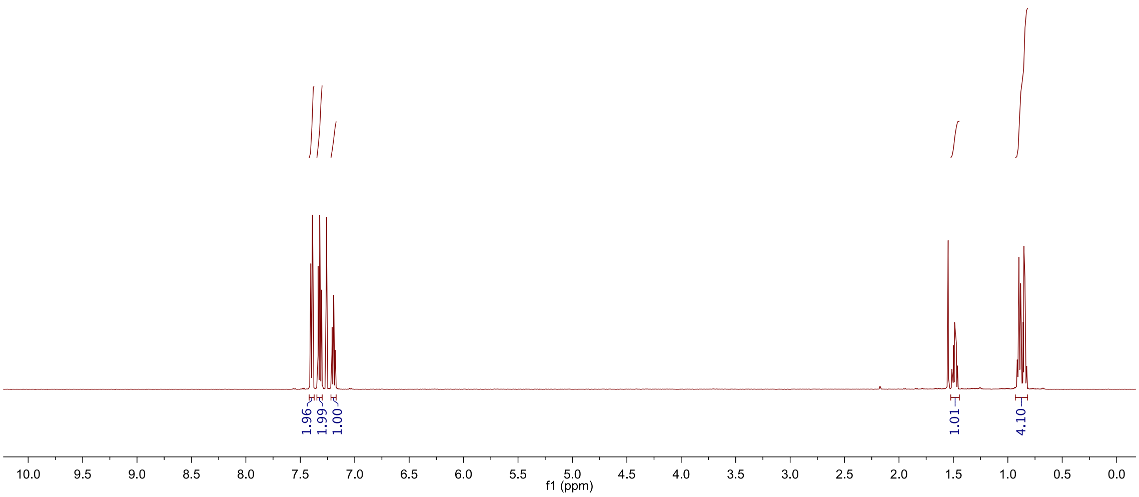

Phenyl(*t*-butylethynyl)sulfane (1f)

01282015-23-doh-db44-N
1H Observe
DB497

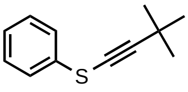

1f

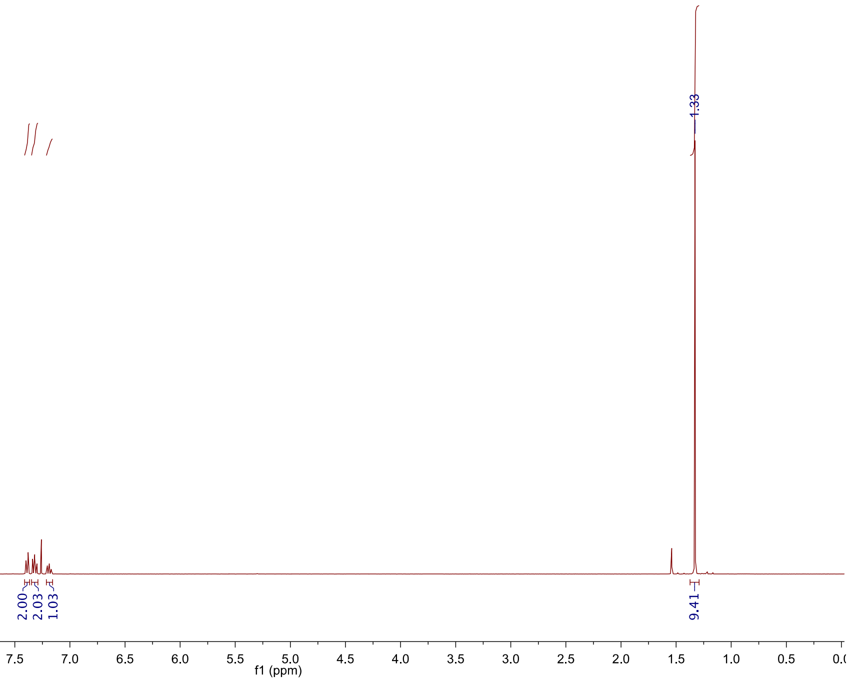

Phenyl(phenylethynyl)sulfane (1g)

02182015-10-doh-db44-F
1H Observe
DB510

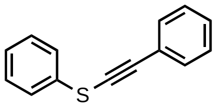

1g

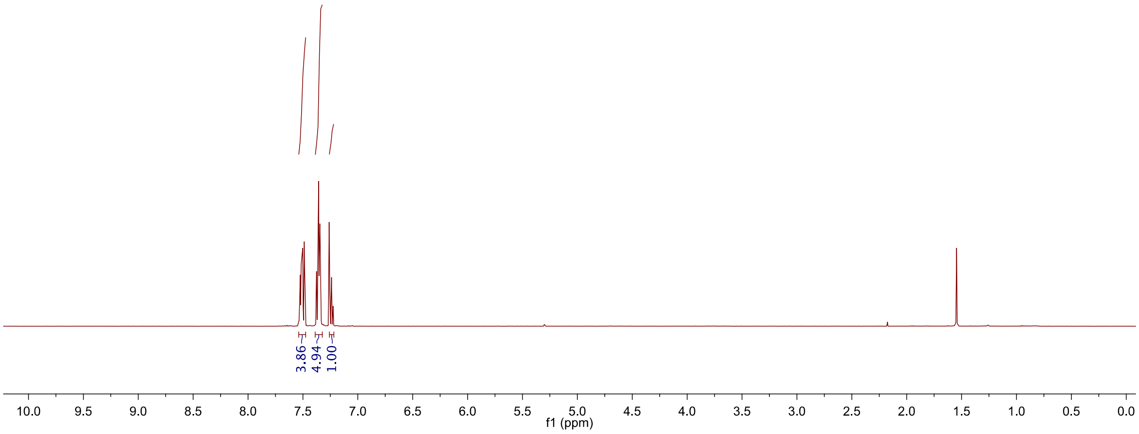

Phenyl(4-methoxyphenylethynyl)sulfane (1h)

02062015-36-doh-db44-A
1H Observe
DB504

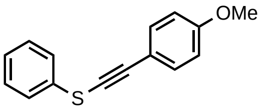

1h

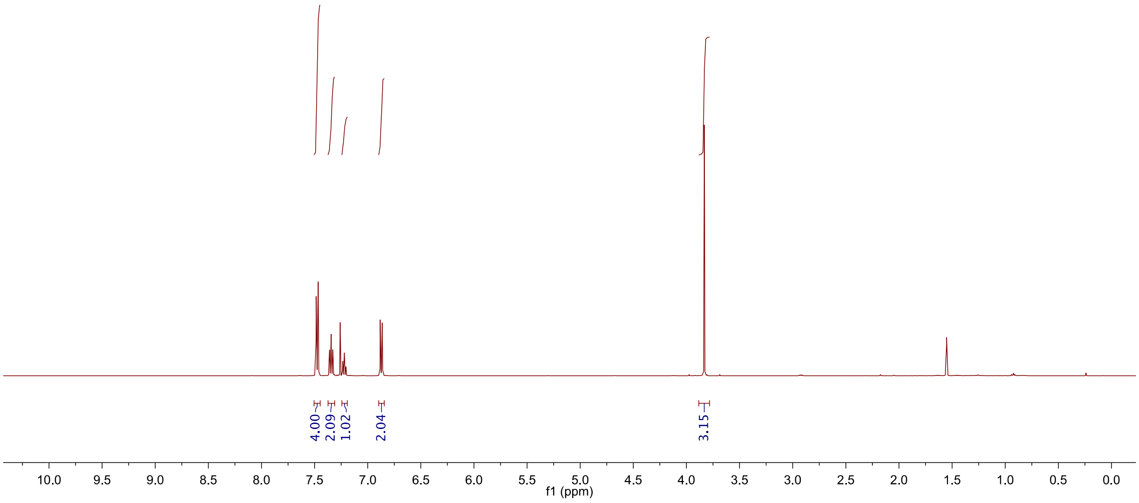

Phenyl(4-nitrophenylethynyl)sulfane (1i)

03092015-20-doh-db44-F
1H Observe
DB521-10

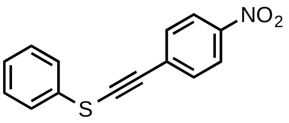

1i

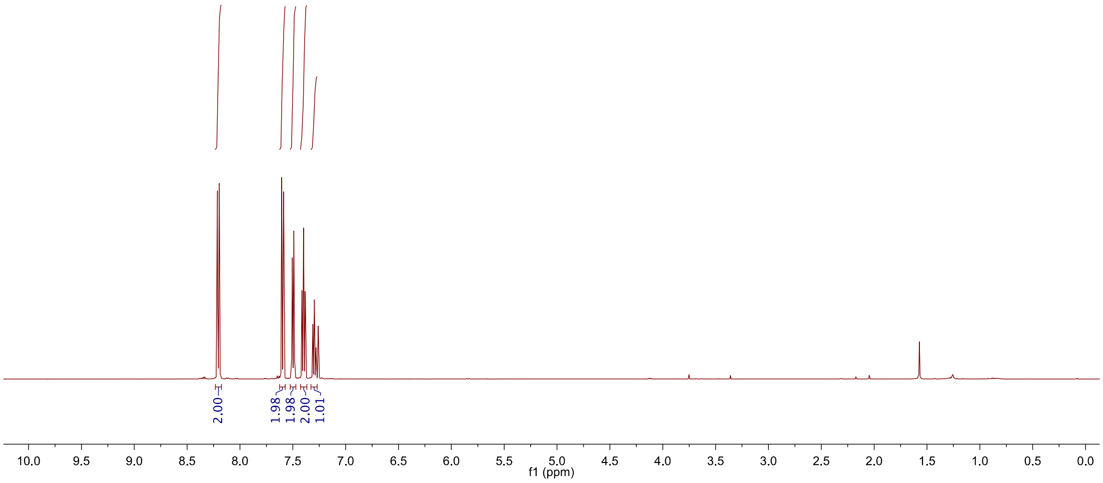

03092015-20-doh-db44-F
13C Observe with 1H decoupling - UDEFT
DB521-10

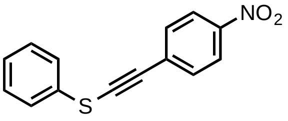

1i

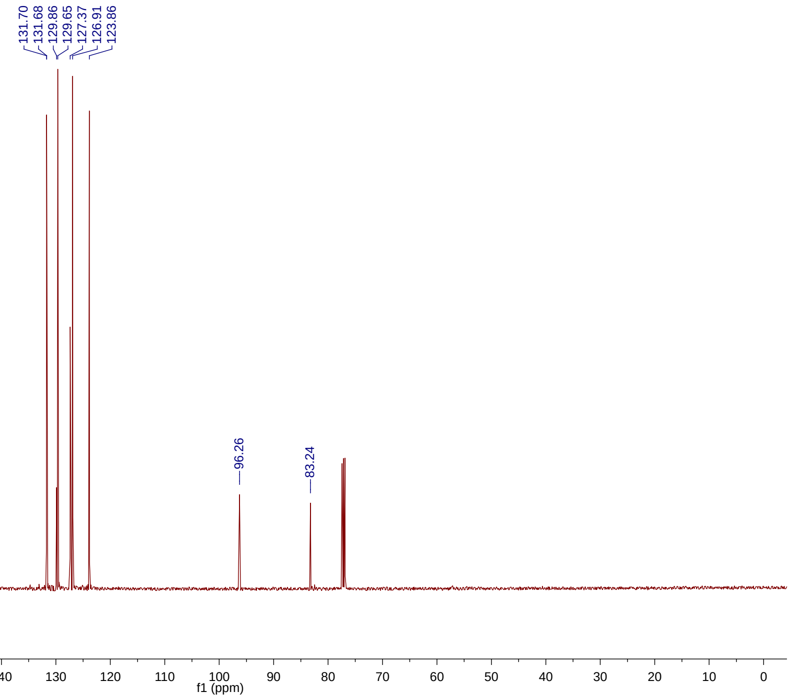

Phenyl(4-trifluoromethylphenylethynyl)sulfane (1j)

03252015-37-doh-db44-F
1H Observe
DB532_5-11

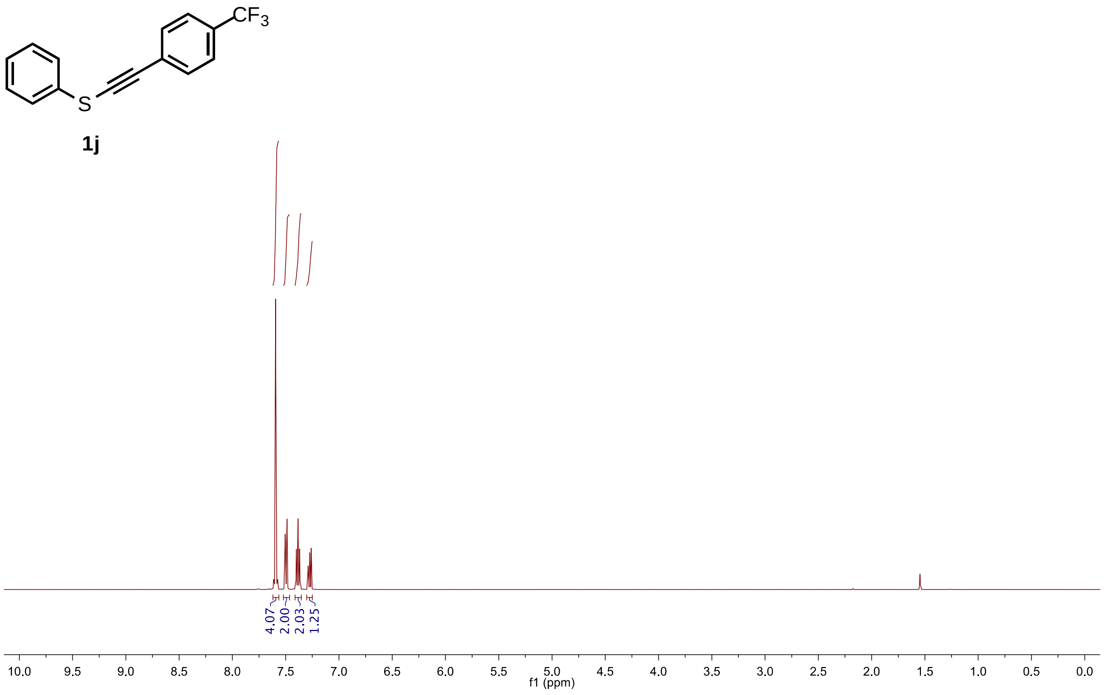

03252015-37-doh-db44-F
19F Observe with 1H decoupling - Full Range SW
DB532_5-11

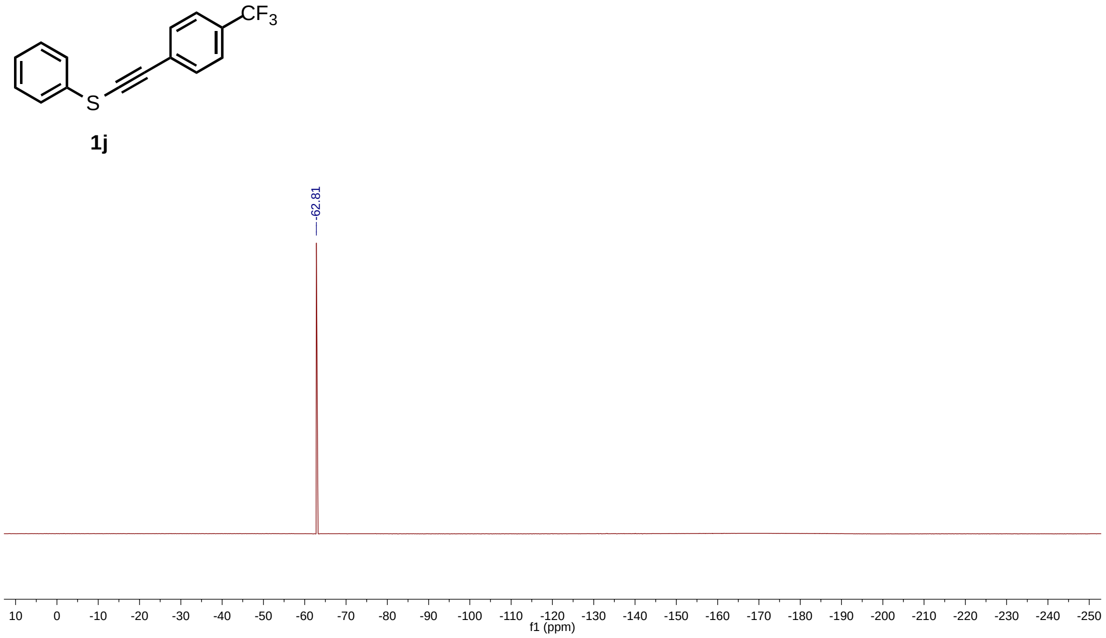

03262015-7-doh-db44-F
13C Observe with 1H decoupling - UDEFT
DB532_5-11_conc

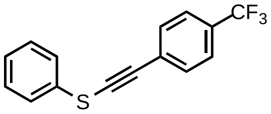

1j

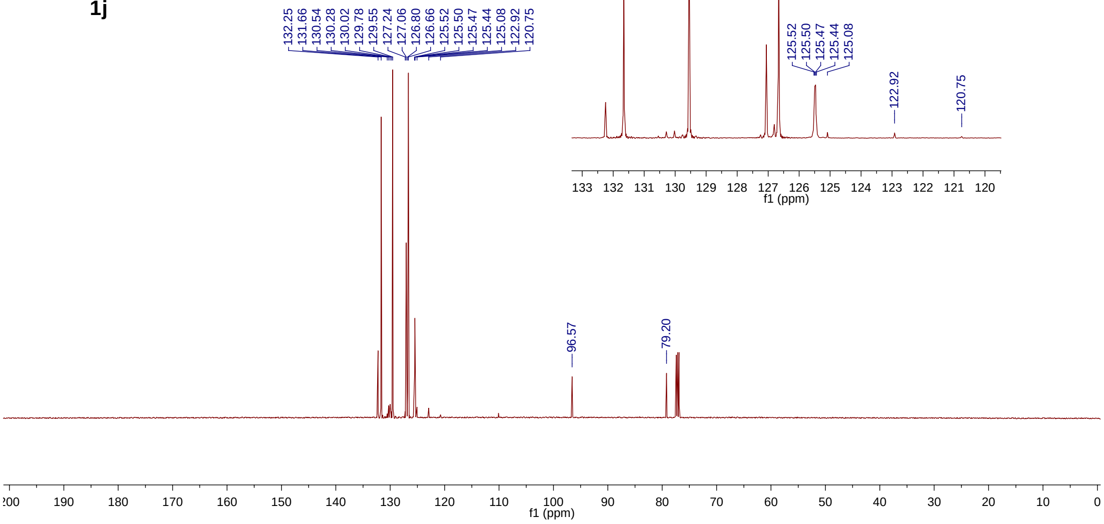

(*Z*)-Benzyl(1-fluorohex-1-en-1-yl)sulfane (2a) and (*E*)-benzyl(1-fluorohex-1-en-1-yl)sulfane (3a)

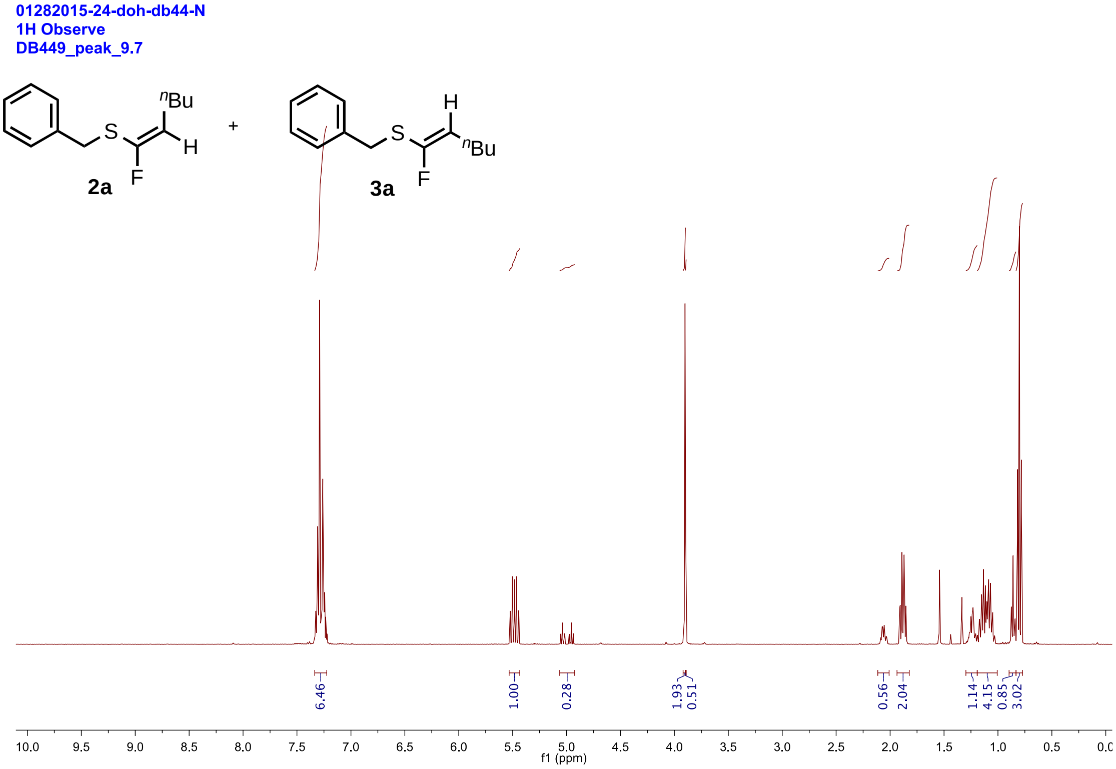

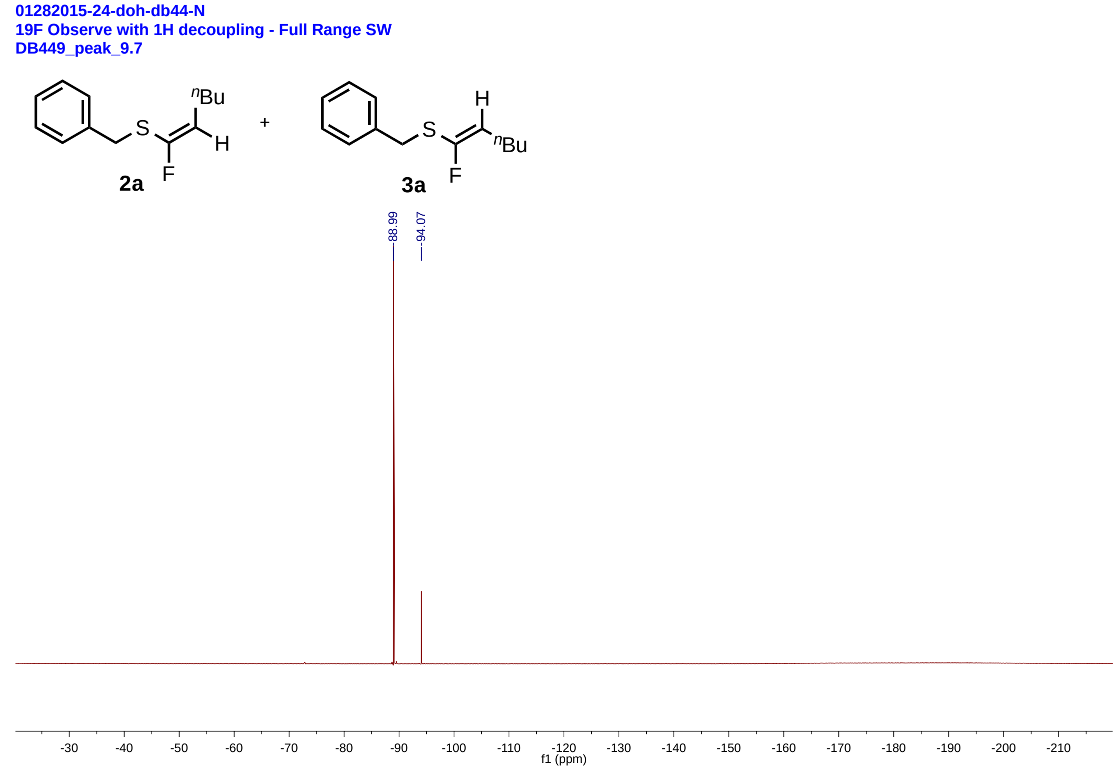

01282015-8-doh-db44-N
13C Observe with 1H decoupling - UDEFT
DB449_peak_9.7

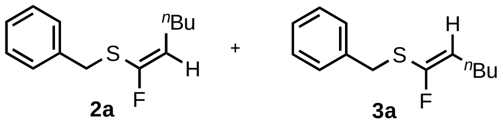

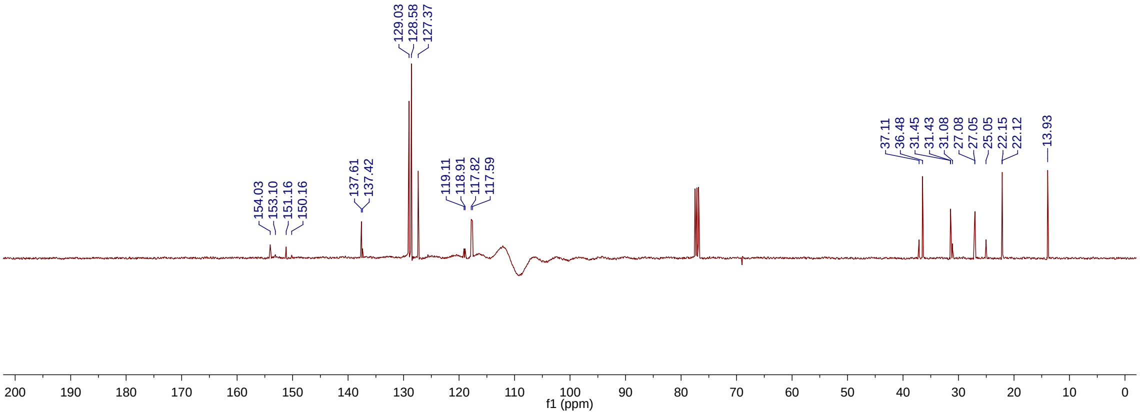

(*Z*)-Benzyl(2-cyclohexyl-1-fluorovinyl)sulfane (2b)

03132015-28-doh-db44-F
1H Observe
DB509-B_HPLC_peak-21.44

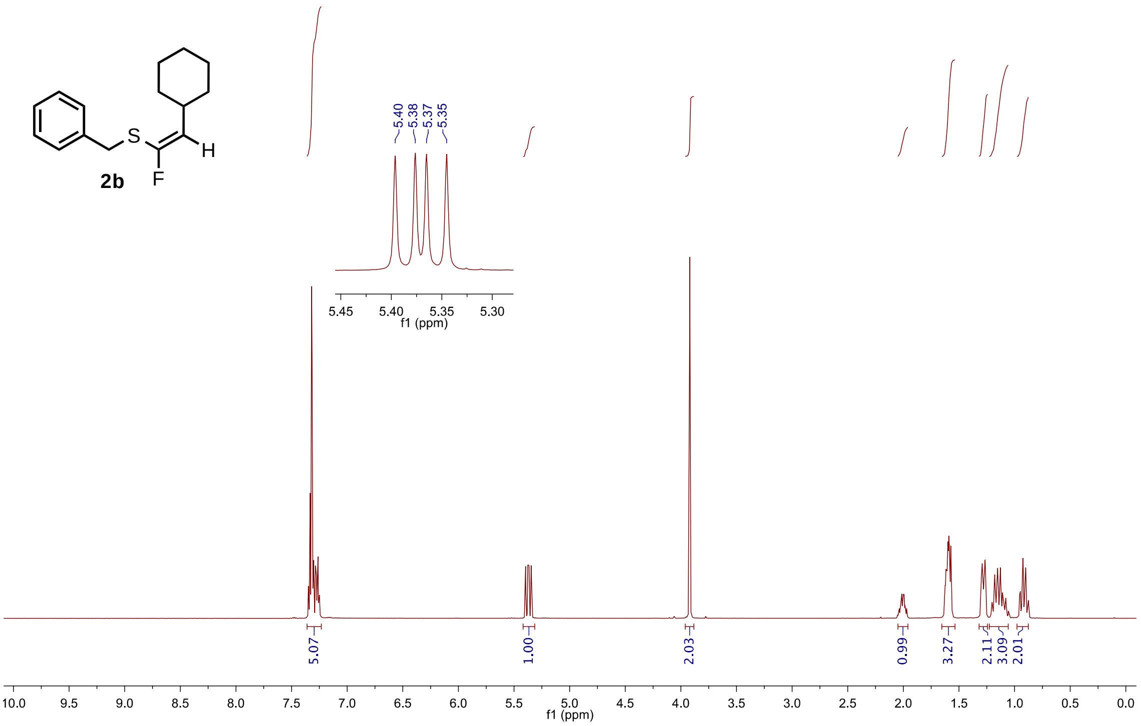

03132015-28-doh-db44-F
19F Observe with 1H decoupling - Full Range SW
DB509-B_HPLC_peak-21.44

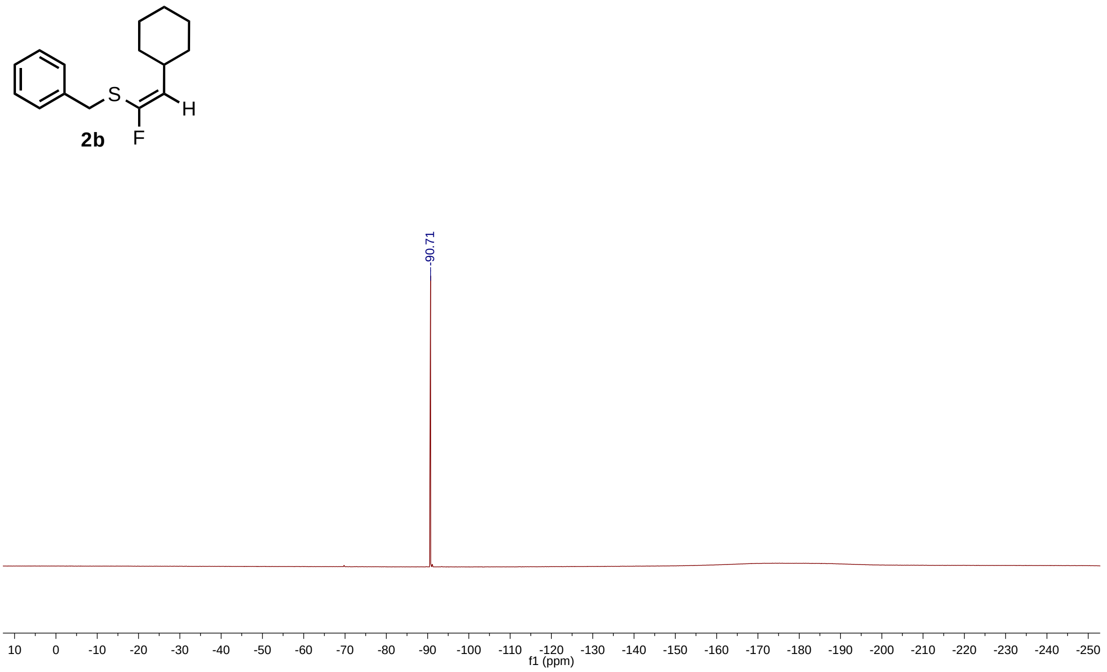

03132015-28-doh-db44-F
13C Observe with 1H decoupling - UDEFT
DB509-B_HPLC_peak-21.44

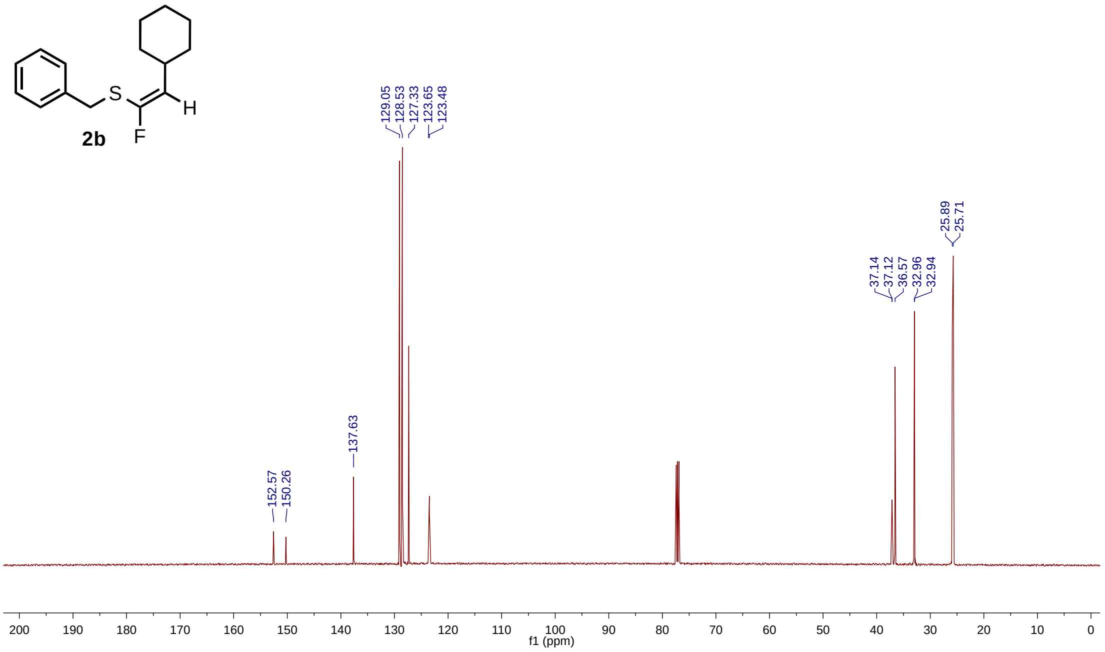

(*E*)-benzyl(2-cyclohexyl-1-fluorovinyl)sulfane (3b)

03132015-29-doh-db44-F
1H Observe
DB509-B_HPLC_peak-23.39

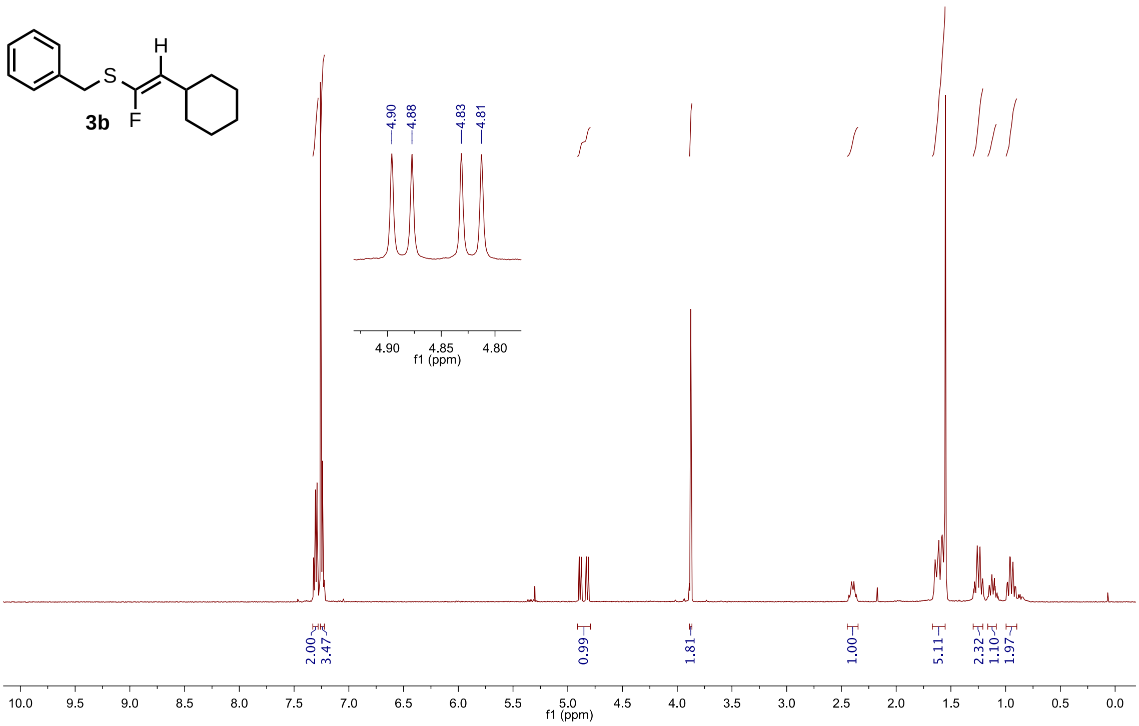

03132015-29-doh-db44-F
19F Observe with 1H decoupling - Full Range SW
DB509-B_HPLC_peak-23.39

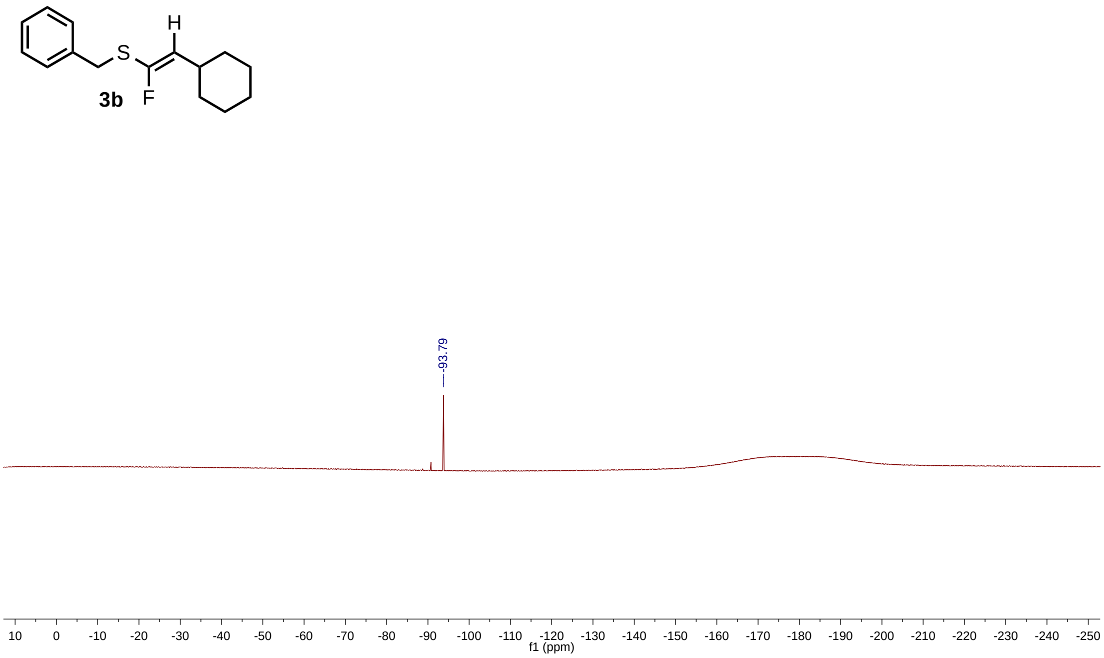

03132015-29-doh-db44-F
13C Observe with 1H decoupling - UDEFT
DB509-B_HPLC_peak-23.39

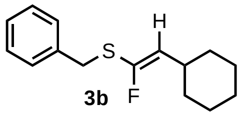

3b

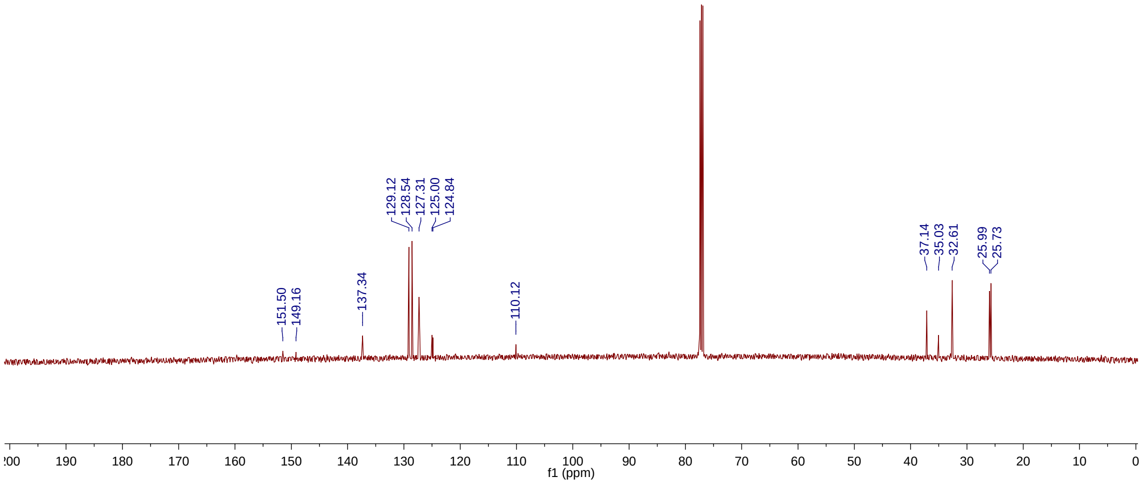

(*Z*)-Benzyl(2-phenyl-1-fluorovinyl)sulfane (2c)

03272015-1-doh-db44-R
1H Observe
DB528-B_11-18

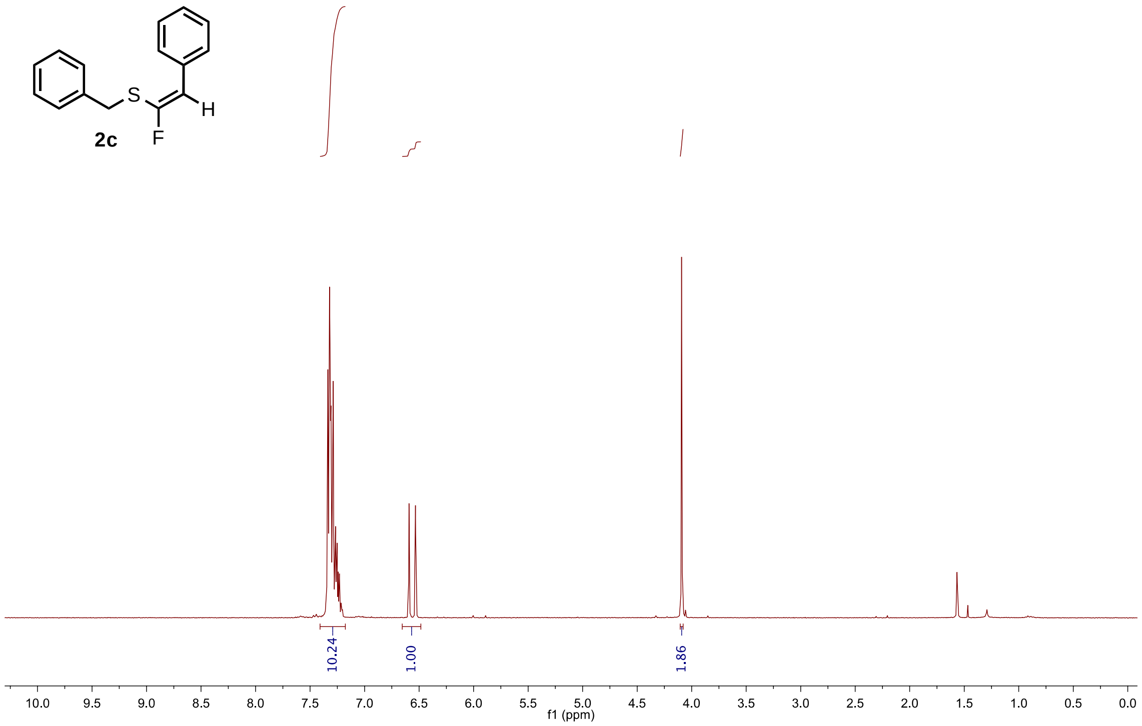

03272015-1-doh-db44-R
19F Observe with 1H decoupling - Full Range SW
DB528-B_11-18

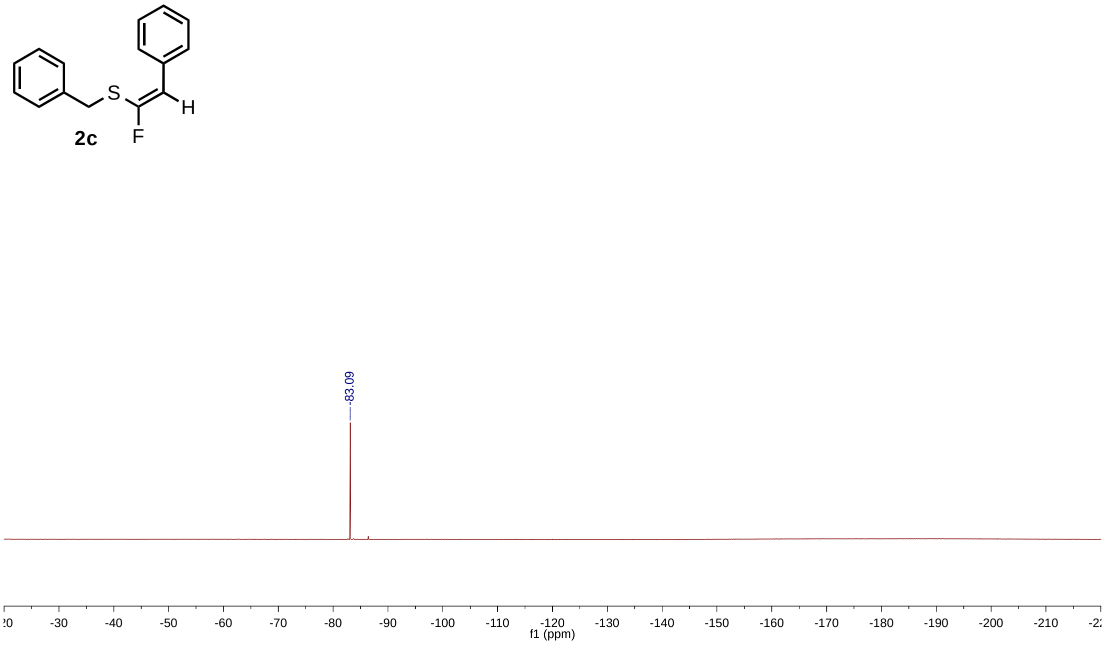

03272015-24-doh-db44-M
13C Observe with 1H decoupling - UDEFT
DB528-B_11-18

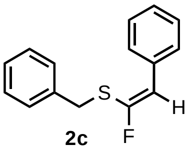
2c

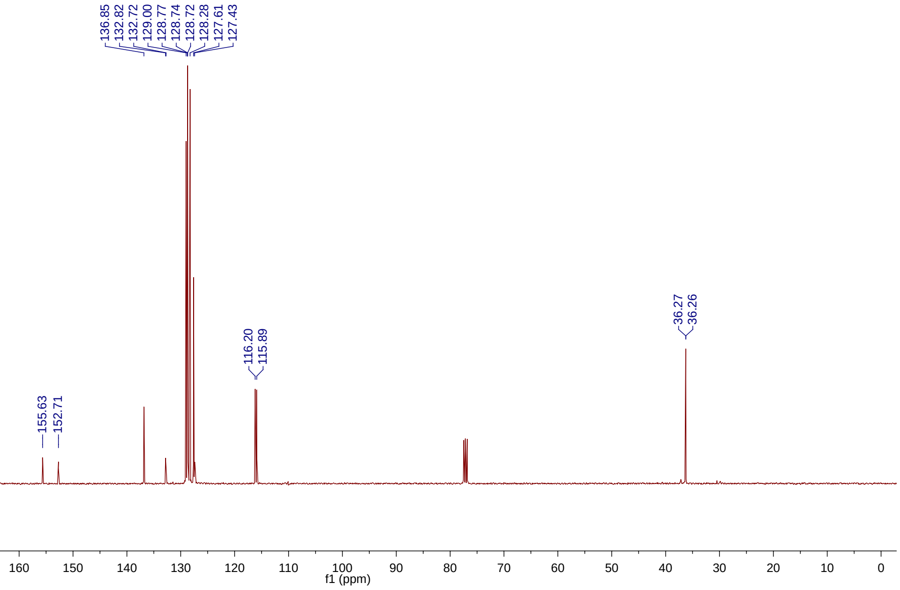

(*Z*)-Cyclohexyl(1-fluoro-2-phenylvinyl)sulfane (2d)

02022015-10-doh-db44-M
1H Observe
DB496-b-5

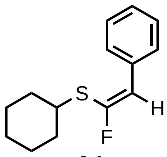

2d

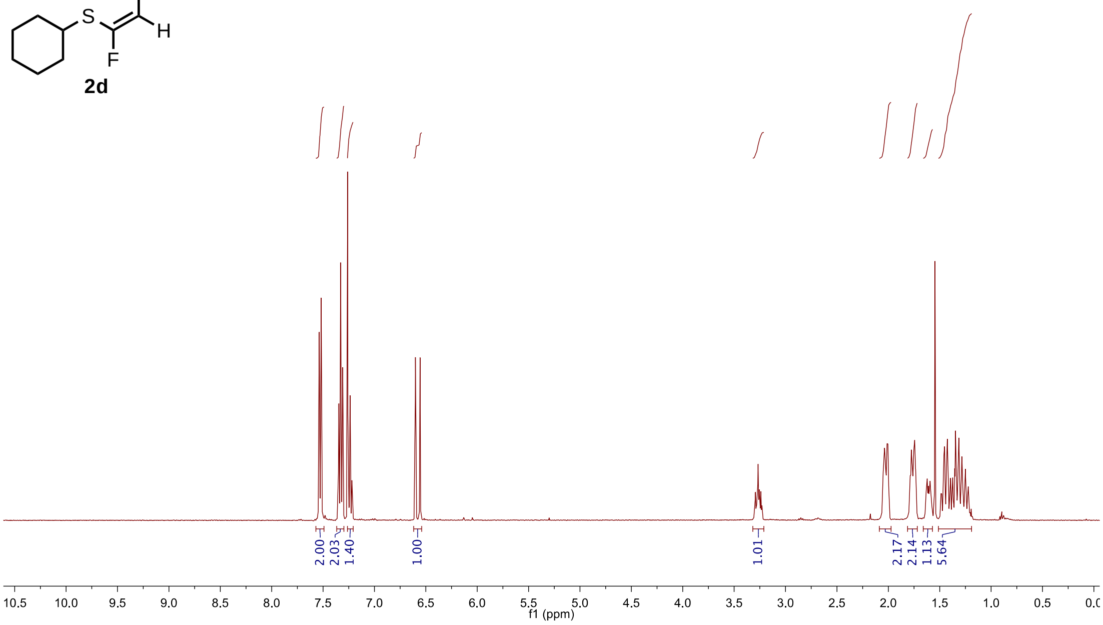

02022015-10-doh-db44-M
19F Observe with 1H decoupling - Full Range SW
DB496-b-5

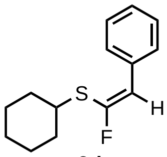

2d

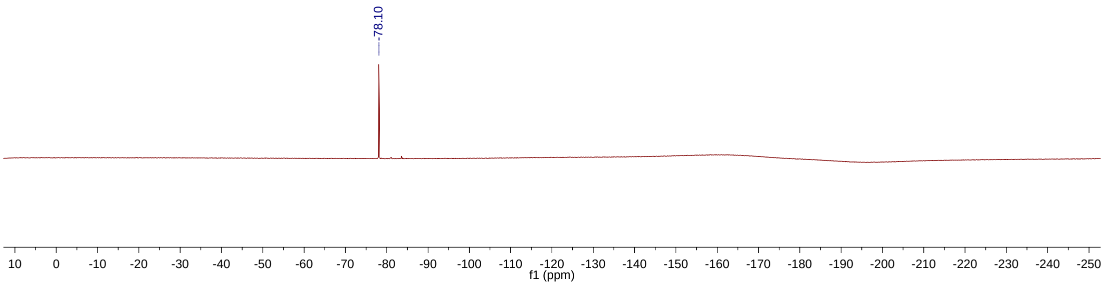

01292015-13-doh-db44-F
13C Observe with 1H decoupling - UDEFT
DB496-B_5

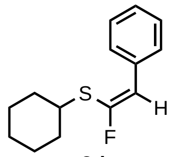

2d

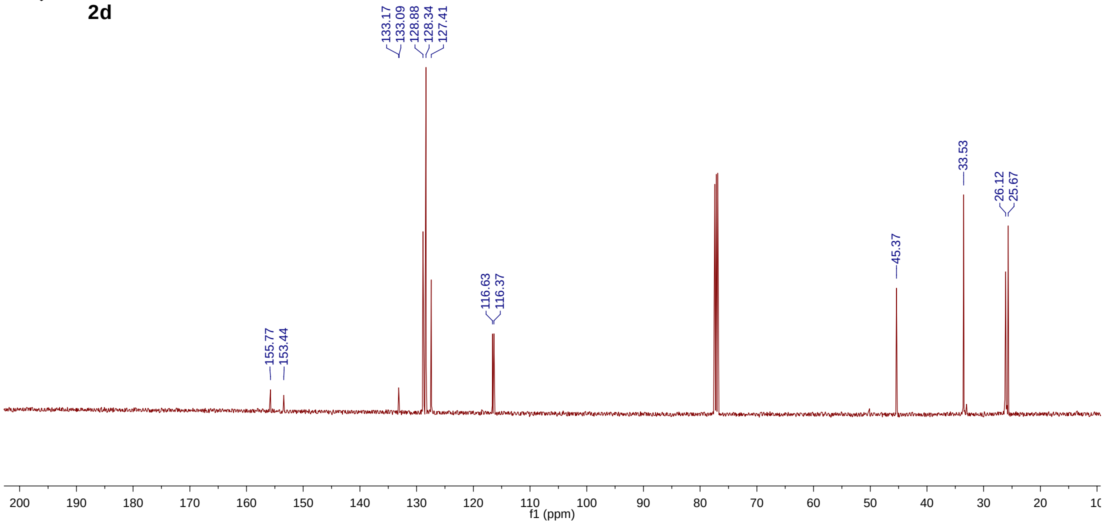

(*Z*)-(2-Cyclopropyl-1-fluorovinyl)(phenyl)sulfane (2e) and (*E*)-(2-cyclopropyl-1-fluorovinyl)(phenyl)sulfane (3e)

03052015-9-doh-db44-F
1H Observe
DB511-B_peak_16.8

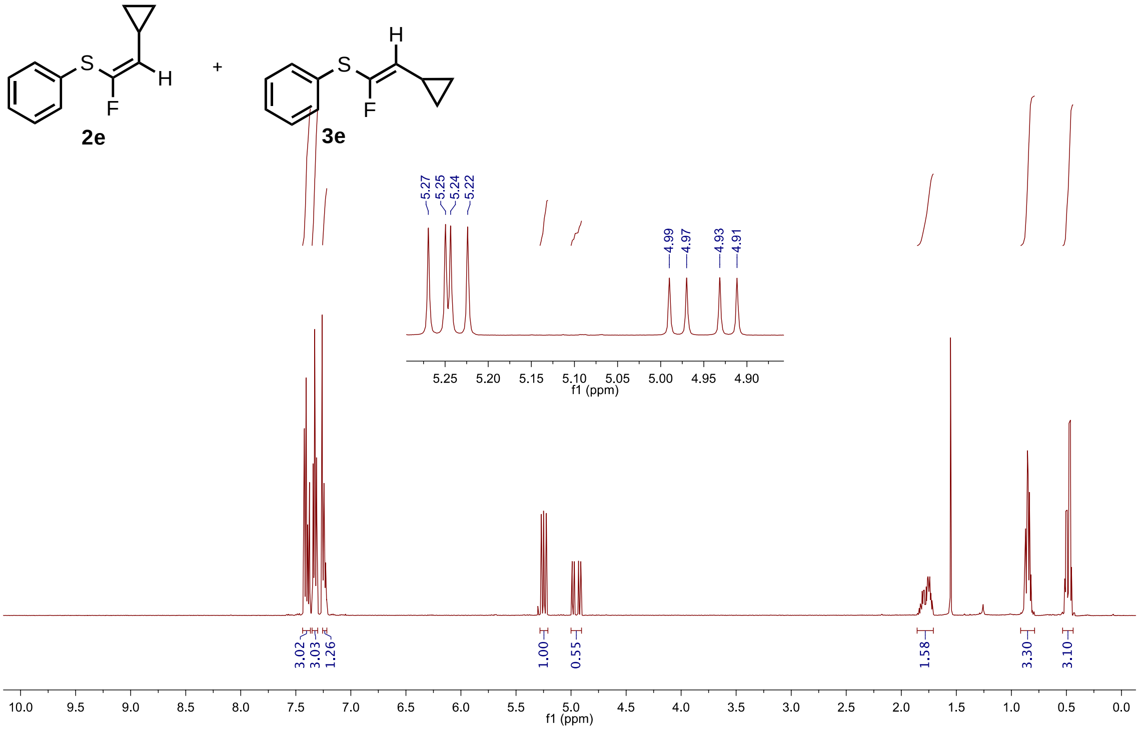

03052015-9-doh-db44-F
19F Observe with 1H decoupling - Full Range SW
DB511-B_peak_16.8

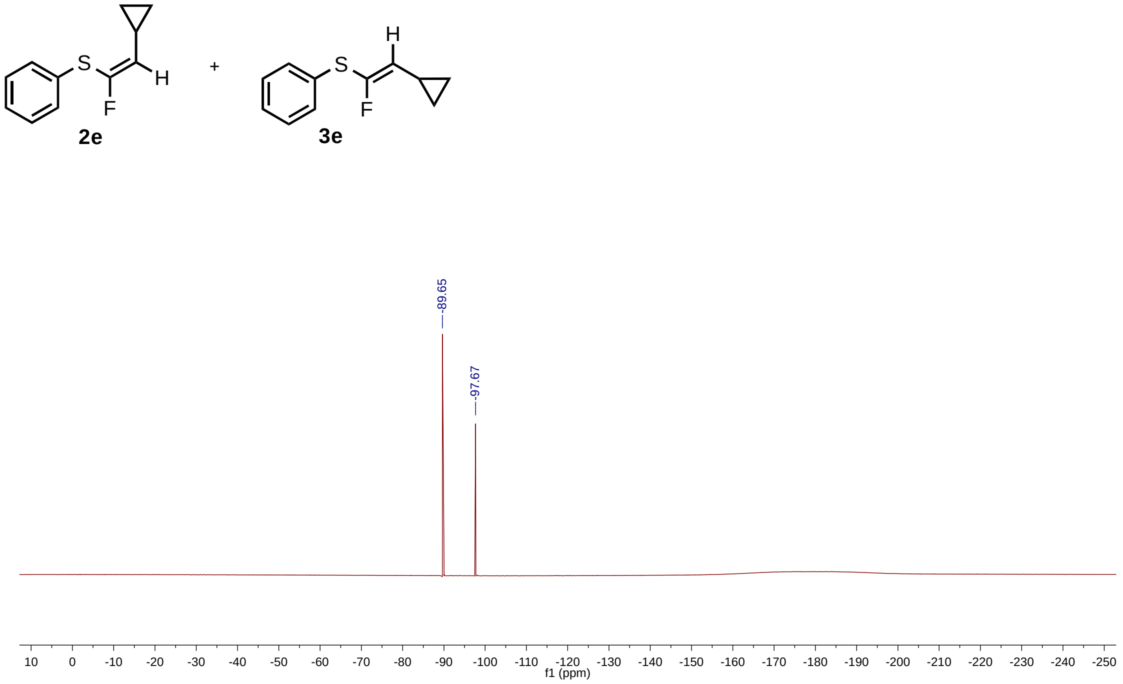

03052015-9-doh-db44-F
13C Observe with 1H decoupling - UDEFT
DB511-B_peak_16.8

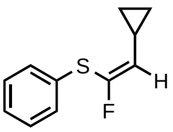

2e

+

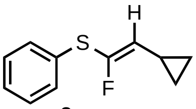

3e

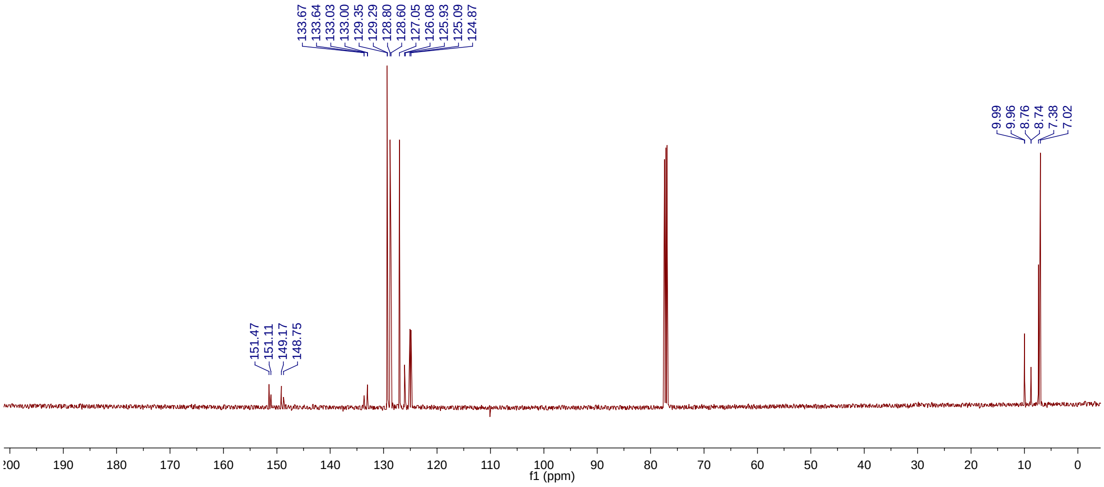

(*Z*)-(1-Fluoro-3,3-dimethylbut-1-en-1-yl)(phenyl)sulfane (2f) and (1,1-difluoro-3,3-dimethylbutyl)(phenyl)sulfane (4f)

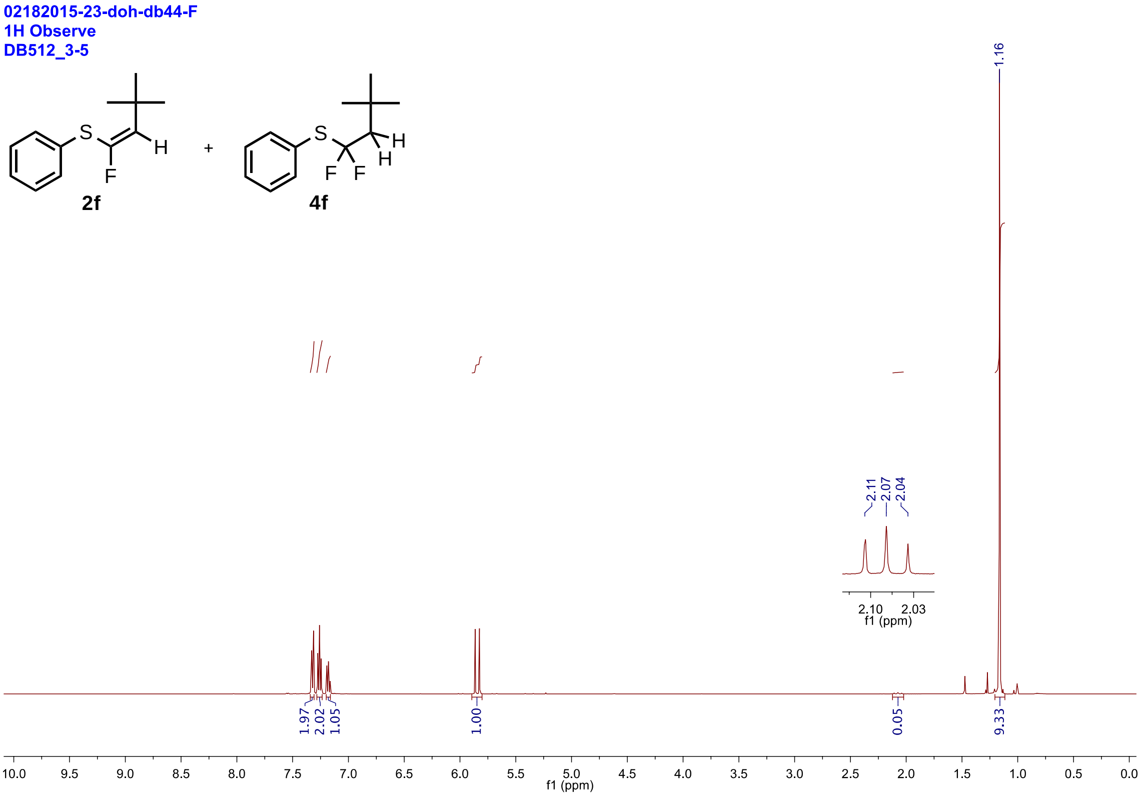

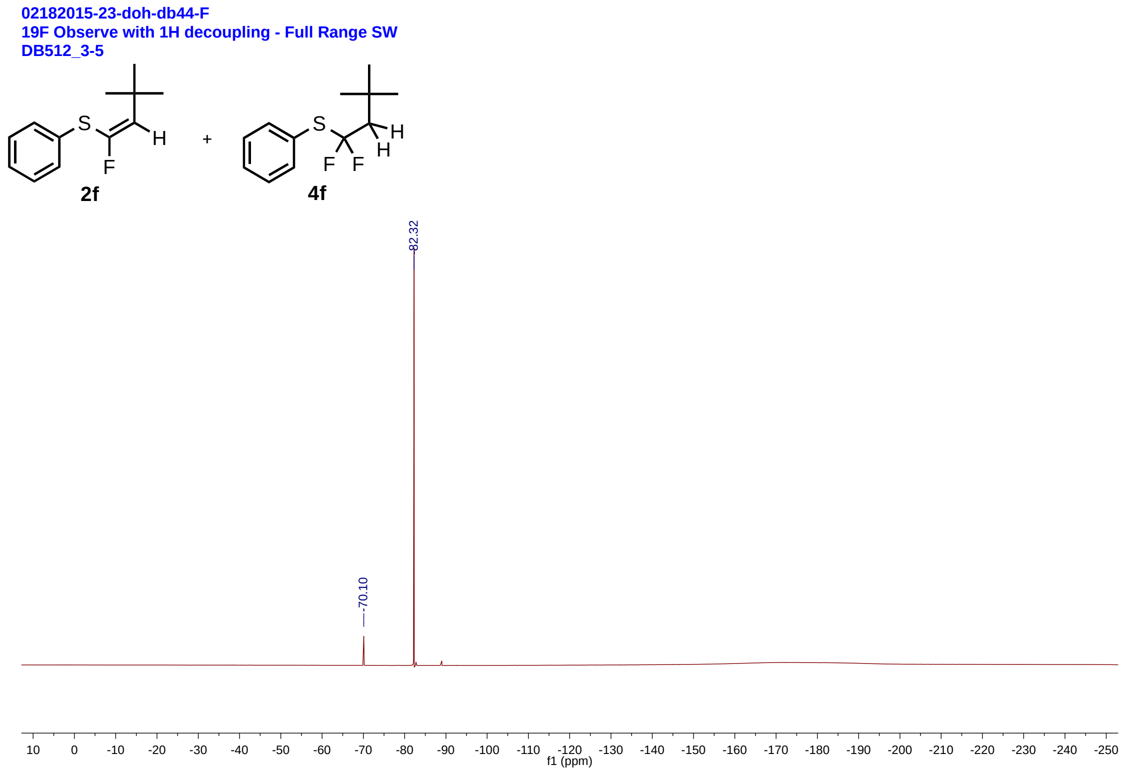

02182015-23-doh-db44-F
19F Observe without 1H decoupling - SW 80 ppm
DB512_3-5

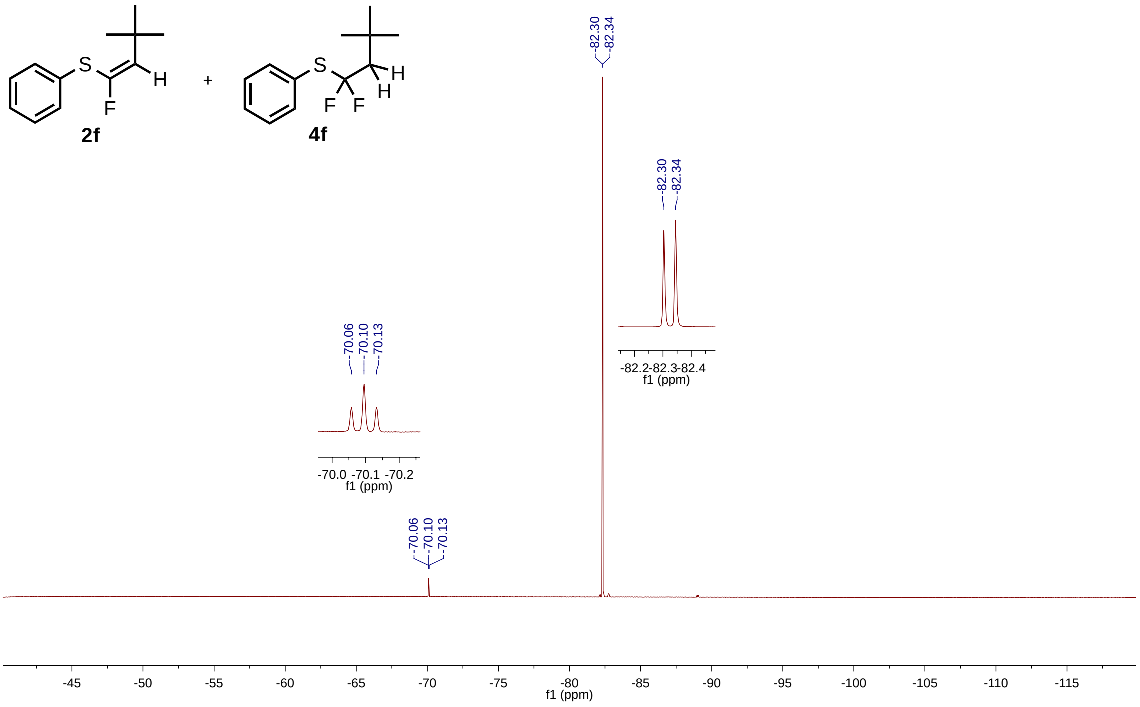

02182015-23-doh-db44-F
13C Observe with 1H decoupling - UDEFT
DB512_3-5

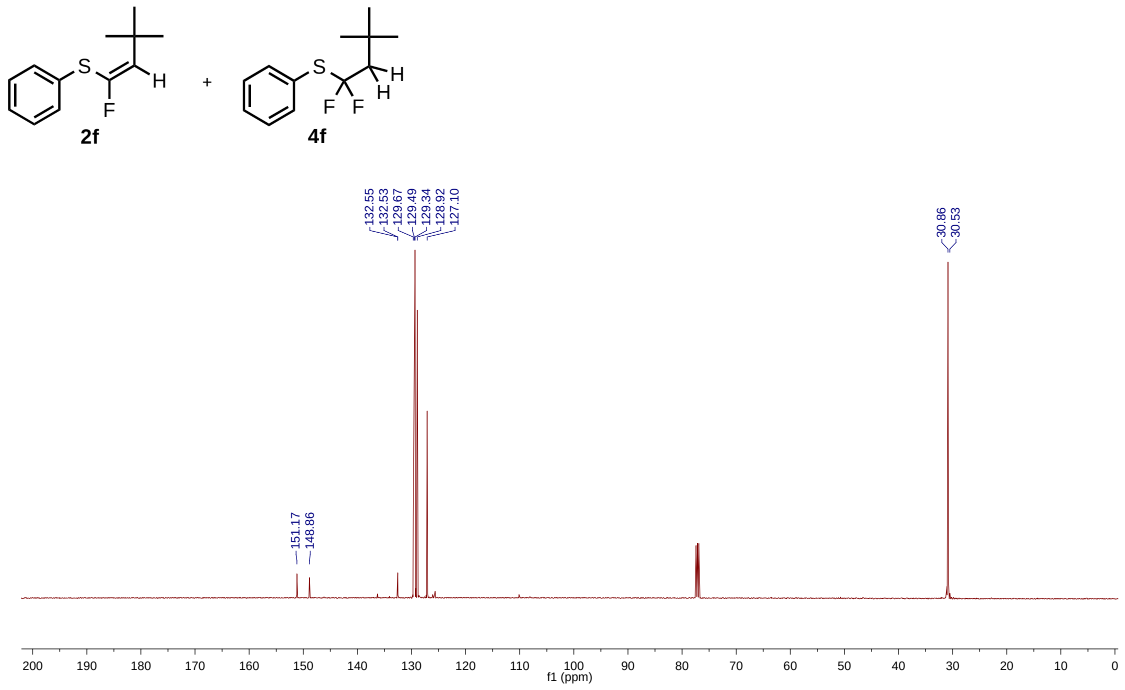

(*Z*)-Phenyl(1-fluoro-2-phenylvinyl)sulfane (2g)

03162015-44-doh-db44-M
1H Observe
DB515-B_HPLC_peak_10.5

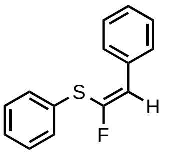

2g

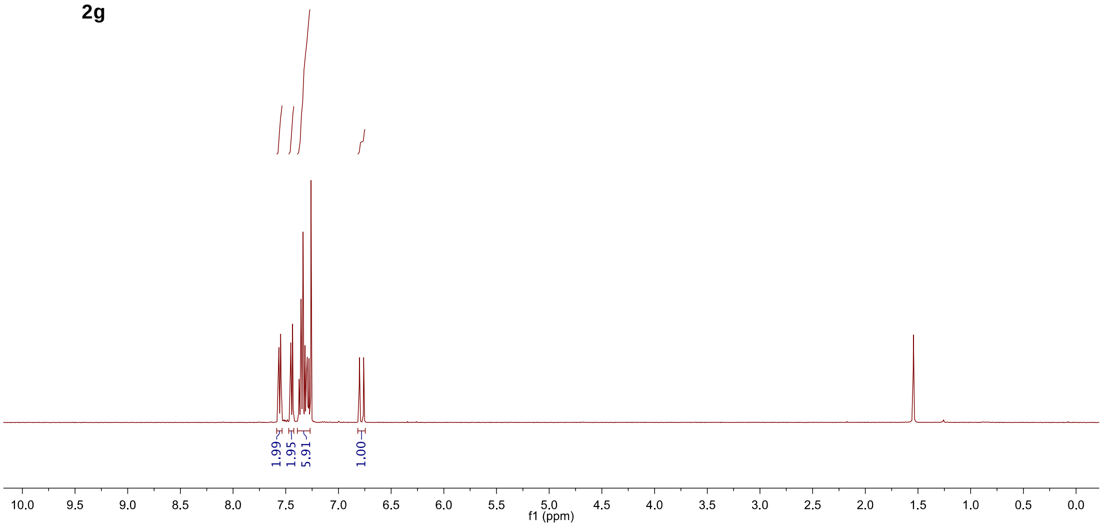

03162015-44-doh-db44-M
19F Observe with 1H decoupling - Full Range SW
DB515-B_HPLC_peak_10.5

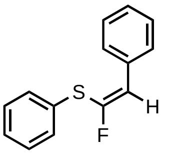

2g

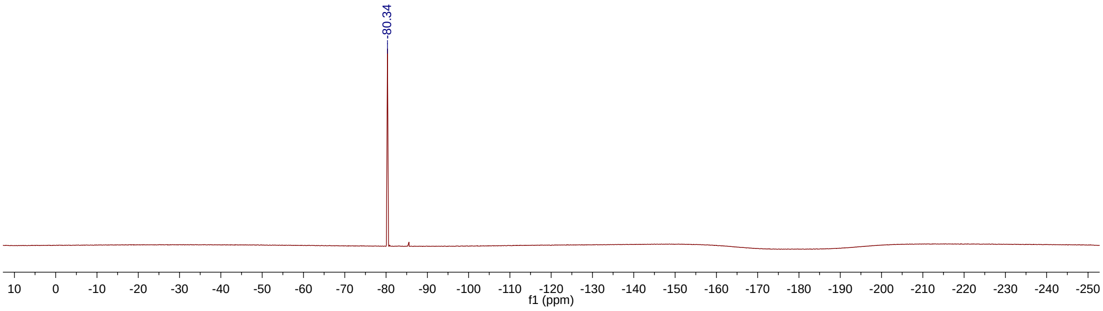

03162015-5-doh-db44-F
13C Observe with 1H decoupling - UDEFT
DB515-B_HPLC_peak_10.5

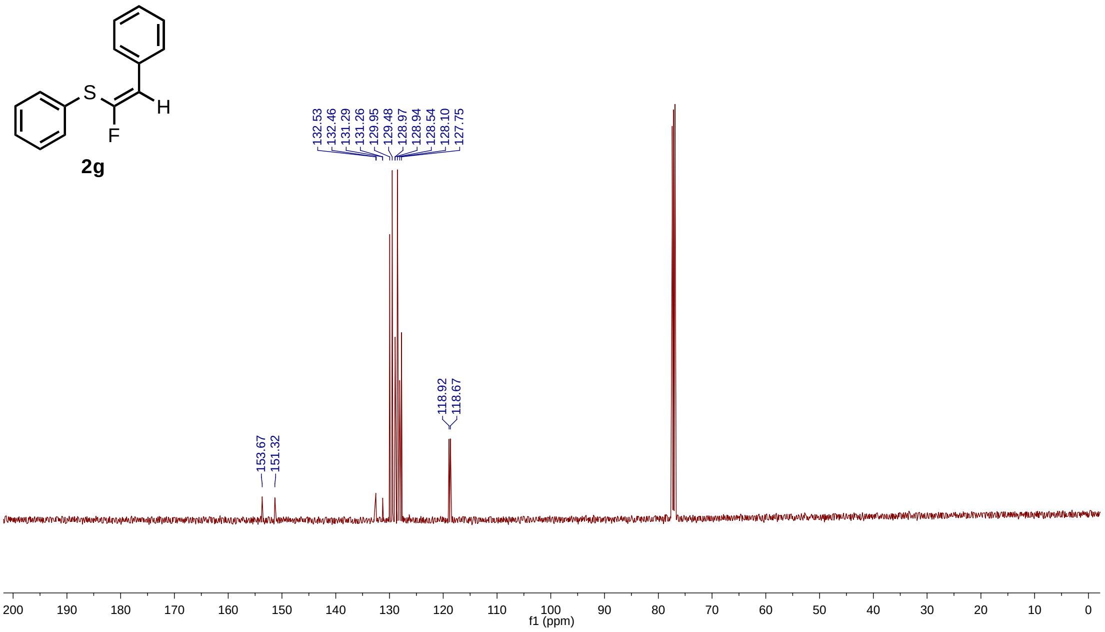

(*Z*)-(1-fluoro-2-(4-methoxyphenyl)vinyl)(phenyl)sulfane (2h)

03022015-21-doh-db44-F
1H Observe
DB514-B_HPLC_peak_12.7

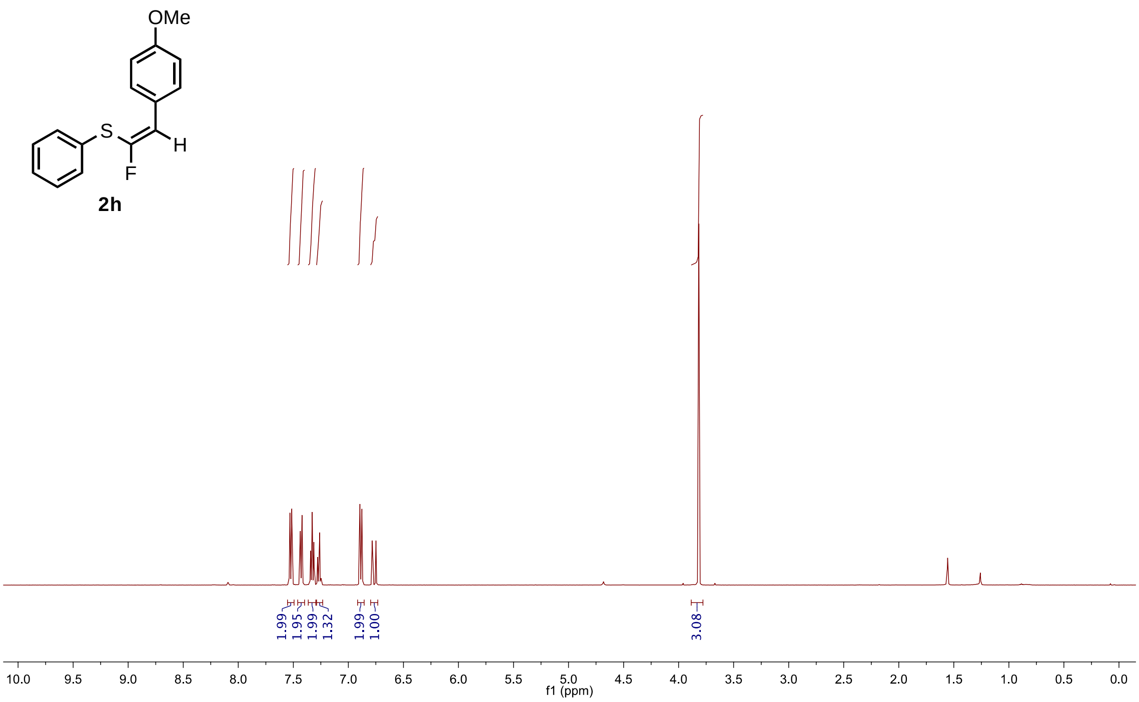

03022015-21-doh-db44-F
19F Observe with 1H decoupling - Full Range SW
DB514-B_HPLC_peak_12.7

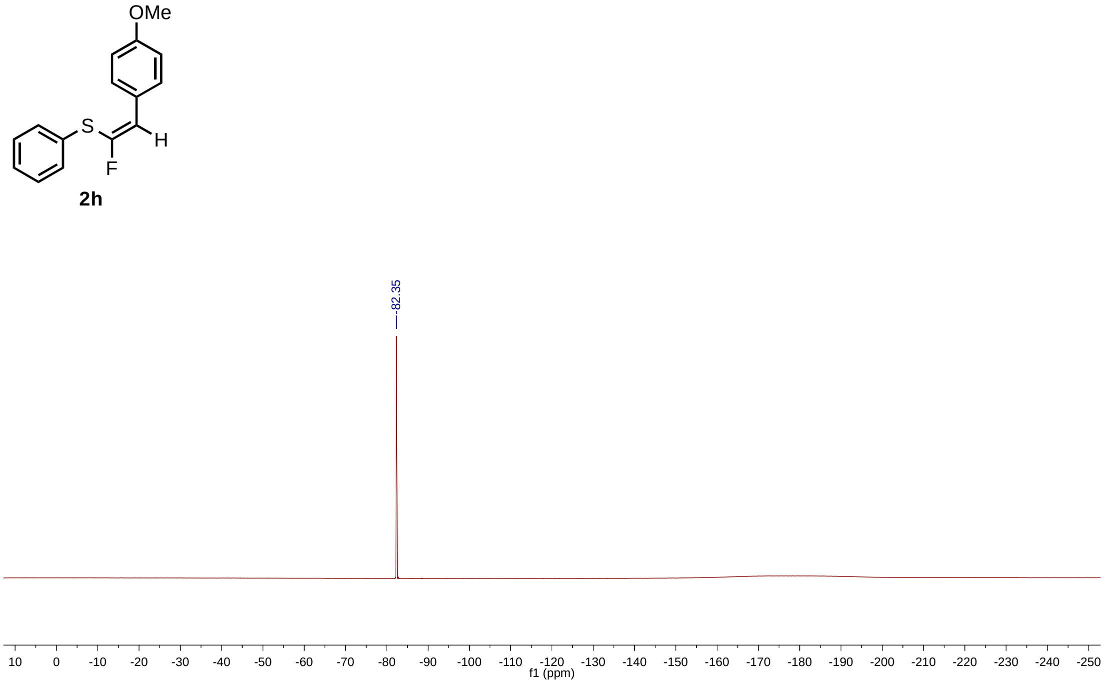

03022015-21-doh-db44-F
13C Observe with 1H decoupling - UDEFT
DB514-B_HPLC_peak_12.7

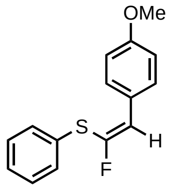

2h

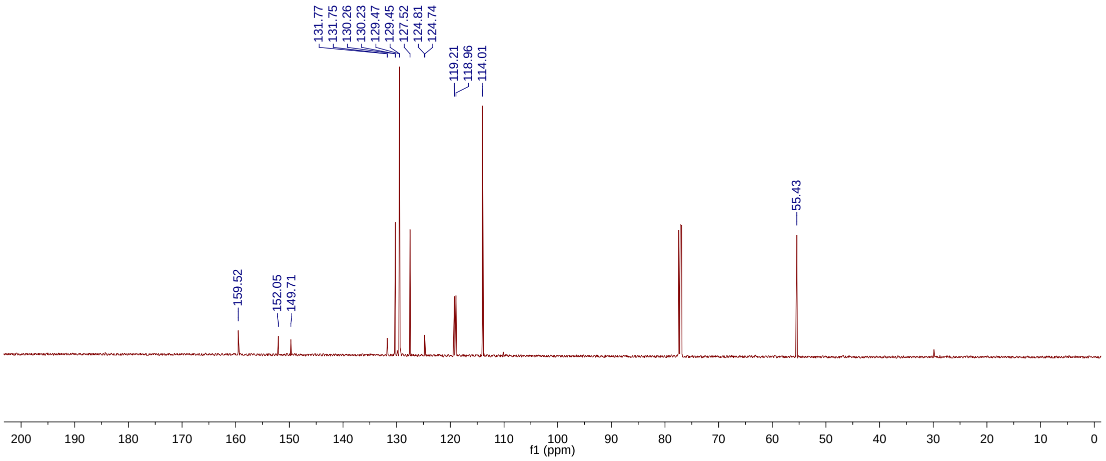

(Z)-(1-fluoro-2-(4-nitrophenyl)vinyl)(phenyl)sulfane (2i)

04072015-3-doh-db44-R
1H Observe
DB527-B_11.7

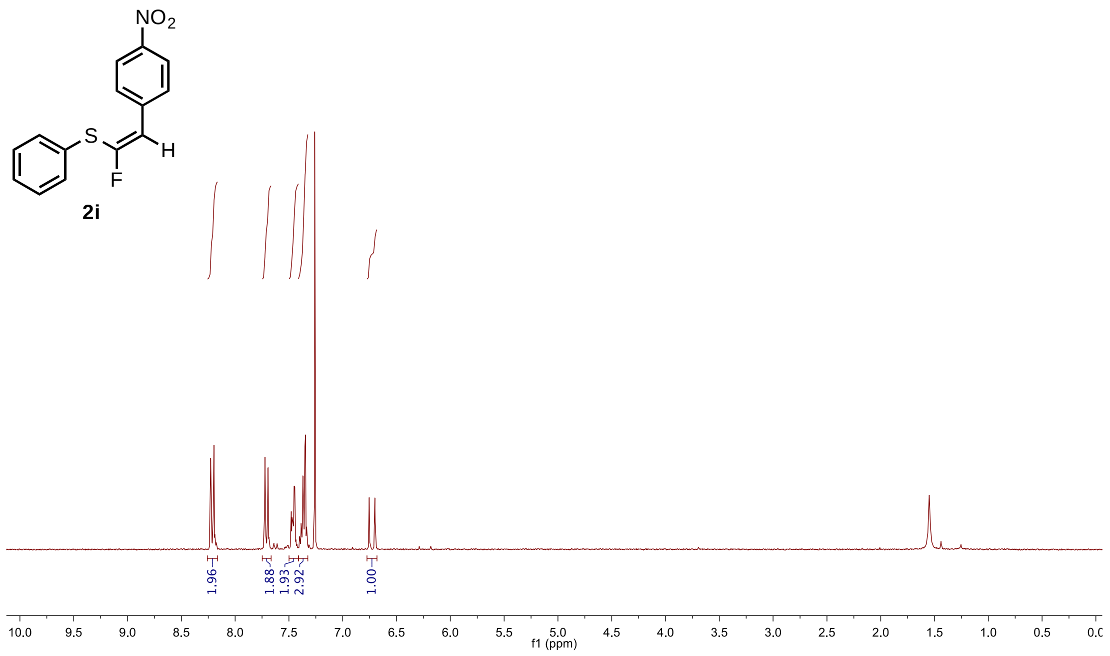

04072015-3-doh-db44-R
19F Observe with 1H decoupling - Full Range SW
DB527-B_11.7

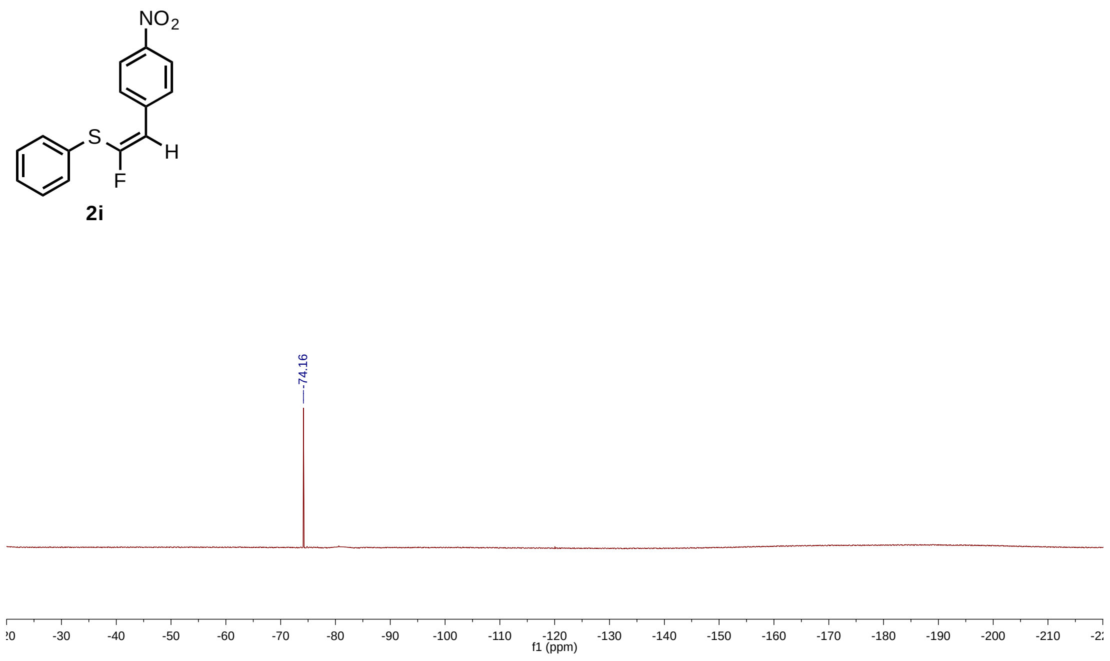

04082015-21-doh-db44-A
13C Observe with 1H decoupling - UDEFT
DB527_B2_peak24

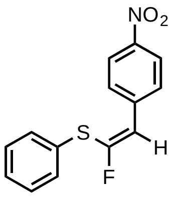

2i

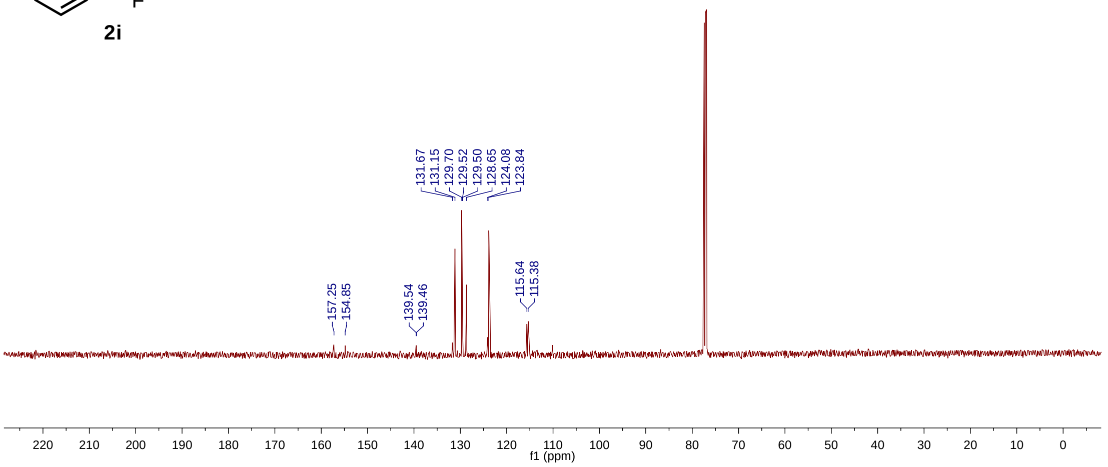

References

1. W. Zheng, F. Zheng, Y. Hong and L. Hu, *Heteroat. Chem.*, **2012**, *23*, 105-110.
2. B. K. Peters, T. Zhou, J. Rujirawanich, A. Cadu, T. Singh, W. Rabten, S. Kerdphon, and P. G. Andersson, *J. Am. Chem. Soc*. **2014**, 136, 16557−16562.
3. N. Riddel, W. Tam; *J. Org. Chem*, **2006,** *71*, 1934 -1937.
4. S. Ding, G. Jia, and J. Sun; *Angew. Chem. Int. Ed.* **2014**, 53, 1877 –1880.
5. M. Voets, M. Smet and W. Dehaen; *J. Chem. Soc., Perkin Trans. 1*, **1999**, 1473-1476.
6. L. Li, F. Wang, C. Ni, and J. Hu; *Angew. Chem. Int. Ed.*; **2013**, *52*, 12390-12394.
